# Timing and expectation of reward: a neuro-computational model of the afferents to the ventral tegmental area

**DOI:** 10.3389/fnbot.2014.00004

**Published:** 2014-01-31

**Authors:** Julien Vitay, Fred H. Hamker

**Affiliations:** ^1^Department of Computer Science, Chemnitz University of TechnologyChemnitz, Germany; ^2^Bernstein Center for Computational Neuroscience, Charité University MedicineBerlin, Germany

**Keywords:** timing, classical conditioning, basal ganglia, dopamine, VTA, amygdala

## Abstract

Neural activity in dopaminergic areas such as the ventral tegmental area is influenced by timing processes, in particular by the temporal expectation of rewards during Pavlovian conditioning. Receipt of a reward at the expected time allows to compute reward-prediction errors which can drive learning in motor or cognitive structures. Reciprocally, dopamine plays an important role in the timing of external events. Several models of the dopaminergic system exist, but the substrate of temporal learning is rather unclear. In this article, we propose a neuro-computational model of the afferent network to the ventral tegmental area, including the lateral hypothalamus, the pedunculopontine nucleus, the amygdala, the ventromedial prefrontal cortex, the ventral basal ganglia (including the nucleus accumbens and the ventral pallidum), as well as the lateral habenula and the rostromedial tegmental nucleus. Based on a plausible connectivity and realistic learning rules, this neuro-computational model reproduces several experimental observations, such as the progressive cancelation of dopaminergic bursts at reward delivery, the appearance of bursts at the onset of reward-predicting cues or the influence of reward magnitude on activity in the amygdala and ventral tegmental area. While associative learning occurs primarily in the amygdala, learning of the temporal relationship between the cue and the associated reward is implemented as a dopamine-modulated coincidence detection mechanism in the nucleus accumbens.

## 1. Introduction

Dopamine (DA) is a key neuromodulator influencing processing and learning in many brain areas, such as the basal ganglia (Bolam et al., 2000; Haber et al., 2000), the prefrontal cortex (Goldman-Rakic et al., 1992; Seamans and Yang, 2004) or the amygdala (Bissière et al., 2003; Pape and Pare, 2010). Dopaminergic neurons in the ventral tegmental area (VTA) and substantia nigra pars compacta (SNc) are phasically activated by unexpected rewards, aversive, salient or novel stimuli (Schultz et al., 1993; Mirenowicz and Schultz, 1994; Horvitz, 2000; Redgrave et al., 2008). During classical conditioning with appetitive rewards (unconditioned stimulus US), cells in VTA gradually show the same phasic activation at the onset of a reward-predicting cue (conditioned stimulus CS), but stop responding to the US when it is fully predicted (Ljungberg et al., 1992; Schultz et al., 1997; Pan et al., 2005). If the reward is expected but omitted, VTA cells show a complete and long-lasting pause (or dip) in firing shortly after the time when the US was expected; if the reward is delivered earlier than expected, VTA cells respond phasically as if it were not predicted, but do not show a dip at the expected time (Hollerman and Schultz, 1998).

This phasic behavior linked to temporal expectation of reward (cancelation of US-related bursts after sufficient training, pause in firing after reward omission, normal bursts if the reward is delivered earlier) indicates that timing mechanisms play an important role in dopaminergic activation. Conversely, DA is well known to influence other timing processes, such as interval timing and duration estimation (Coull et al., 2011; Kirkpatrick, 2013). Reward magnitudes can alter the estimation of time in peak-interval procedures (where the consumatory response rate in anticipation of an expected reward usually peaks at the learned time), either leftward (the temporal estimation is earlier than what it really is) or rightward (later), the same effect being observed with elevated or reduced DA activity in SNc/VTA (Galtress and Kirkpatrick, 2009). Understanding the interaction between the reward/motivational systems and timing processes is therefore of critical importance (Galtress et al., 2012; Kirkpatrick, 2013). The objective of this article is to propose a neuro-computational model incorporating the afferent structures to the dopaminergic system which are involved in appetitive conditioning and to better describe the neural mechanisms leading to the observed temporal behavior of dopaminergic neurons.

The *temporal difference* (TD) algorithm originally proposed by Sutton and Barto (1981) has become an influential model linking DA activity to timing mechanisms (Montague et al., 1996; Schultz et al., 1997). TD is a unitary mechanism describing DA activity as a reward-prediction error: the difference between the reward expectation in a given state and the actually received reward. Early implementations of TD have used serial-compound representations to represent the presence of a stimulus over time, allowing to reproduce some aspects of DA firing during classical conditioning by chaining backwards in time the association between the CS and the US (Suri and Schultz, 1999, 2001). This would predict a progressive backward shift of the US-related burst during learning, what is experimentally not the case, as the CS- and US-related bursts gradually increase and decrease with learning, respectively. Different temporal representations of the stimuli can overcome this issue. Using long eligibility traces (TD(λ), Sutton and Barto, 1998), the algorithm can be turned into a more advanced associative learning rule to better fit the experimental data (Pan et al., 2005). Using a series of internal microstimuli growing weaker and more diffuse over time also allows to overcome this problem as well as to better capture DA activity when a reward is delivered earlier as predicted (Ludvig et al., 2008). An adequate temporal representation of stimuli can even be learned in an unsupervised manner through the use of long short-term memory (LSTM) networks (Rivest et al., 2010, 2013). Overall, TD-based algorithms are an important model of DA activity, both because of their mathematical elegance and predictive power, and are widely used for explaining experimental data in decision-making (for example Daw et al., 2005; Samejima and Doya, 2007; Rao, 2010) and in neurorobotical systems (for example Sporns and Alexander, 2002; Krichmar, 2013).

Other models have been proposed to better explain the experimental data while improving the biological plausibility. One important class of models are the *dual-pathway* models, which hypothesize that the different components of DA activation are computed in segregated brain areas projecting onto the SNc/VTA (Brown et al., 1999; O'Reilly et al., 2007; Tan and Bullock, 2008; Hazy et al., 2010). These models share some common assumptions about the mechanisms, although the putative brain areas may differ: reward delivery provokes DA bursts through glutamatergic projections from the pedunculopontine nucleus (PPTN); the conditioning strength of the CS is first acquired in the amygdala or the ventral striatum and then transferred to the DA cells either directly or through PPTN; the cancelation of predicted US bursts and the dips at reward omission originate from the striosomes of the dorsal or ventral striatum which project inhibitorily to VTA/SNC. The origin of the latter signals, which have a strong temporal component, differ however between these models. The models by Brown et al. (1999) and Tan and Bullock (2008) consider that cells in the striosomes of the dorsal and ventral striatum implement an *intracellular spectral timing* mechanism (Grossberg and Schmajuk, 1989), where each cell in these populations has an internal calcium variable peaking at a given time after the CS onset and emits delayed spikes. The cell being active at reward delivery (signaled by the DA burst) becomes representative of the elapsed duration. The models by O'Reilly et al. (2007) and Hazy et al. (2010) more abstractly consider a ramping function peaking at the estimated reward delivery time, and originating from the cerebellum. How this timing signal from the cerebellum is adapted to different CS-US intervals is not explicitely modeled.

Spectral timing mechanisms have been observed in the cerebellum (Fiala et al., 1996) but not in the striatum. The cerebellum is critically involved in aversive conditioning such as the rabbit eye-blink conditioning (Christian and Thompson, 2003; Thompson and Steinmetz, 2009), but its involvement in appetitive conditioning is still unknown (see Martin-Soelch et al., 2007). Moreover, the intracellular mechanisms necessary for spectral timing may not efficiently apply to the supra-second range used in most appetitive conditioning experiments (Matell and Meck, 2004; Coull et al., 2011). The neural substrate of temporal learning in dual-pathway models of the dopaminergic system needs further investigation.

The goal of the present article is to investigate how far dual-pathway models of reward prediction can be adapted to take into account the recent wealth of experiments investigating timing processes in the brain (Coull et al., 2011; Kirkpatrick, 2013). Although most of them focus on operant conditioning, they point at a critical role of the striatum in learning supra-second durations. One of the most biologically plausible model of interval timing to date is the *Striatal-Beat Frequency* model (Matell and Meck, 2000, 2004; Lustig et al., 2005), which proposes that striatal neurons act as coincidence detectors, reacting maximally when a series of cortical oscillators, synchronized at CS onset, is in a particular configuration. We propose that a similar mechanism is used to control the temporal behavior of dopaminergic cells during appetitive conditioning.

We present a neuro-computational model incorporating many areas involved in appetitive conditioning and reward processing, including the amygdala, the ventral basal ganglia and various forebrain nuclei projecting to VTA/SNc. It focuses on the phasic components of dopaminergic activation and reproduces the behavior of VTA cells during conditioning, especially with respect to different reward magnitudes, reward omission or earlier delivery. However, it is not designed to address the tonic component of DA activation, nor the observed dependency of VTA firing on reward probability (Fiorillo et al., 2003). From the computational point of view, it provides a robust and autonomous mechanism to learn CS-US associations with variable durations.

## 2. Materials and methods

### 2.1. Neurobiological assumptions

#### 2.1.1. Appetitive delay conditioning

The proposed model of dopaminergic activation during conditioning is restricted in its current form to appetitive conditioning, where the US is a physical reward such as food. Aversive conditioning, where the US is a painful stimulation or a frightening stimulus, engages similar structures—in particular, the amygdala, the ventral striatum and the dopaminergic system (LeDoux, 2000; Delgado et al., 2008; Matsumoto and Hikosaka, 2009)—but the model does not aim at reproducing these effects. The cerebellum plays a much more important role in aversive than in appetitive conditioning (Thompson and Steinmetz, 2009). There is still a debate on whether the same DA cells are activated by appetitive and aversive rewards or if two segregated populations exist (Lammel et al., 2012).

The model is also limited to delay conditioning, where the CS is still physically present (visually or auditorily) when the US arrives. Trace conditioning introduces a temporal gap between the CS and the US. In this case, even small intervals can impair the learned association strength (Raybuck and Lattal, 2013). The medial prefrontal cortex and hippocampus are necessary for trace conditioning to take place, but not delay conditioning (Ito et al., 2006; Walker and Steinmetz, 2008; Wu et al., 2013). This indicates that working memory processes (either through sustained activation or synaptic traces) are involved in trace conditioning, what is not covered by this model. Some TD-based implementations are able to learn both delay and trace conditioning tasks: the model of Ludvig et al. (2008) uses a series of temporal basis functions to represent the trace of the stimuli, what allows the TD algorithm to associate reward delivery to the correct timing. The model of Rivest et al. (2010, 2013) learns an adequate temporal representation for both CS and US using a long short-term memory (LSTM) network (Hochreiter and Schmidhuber, 1997) which is able to fill an eventual gap between the CS and the US.

Dual-pathway models focus mainly on delay conditioning: Brown et al. (1999) propose that a bistable representation of CS information, mimicking the sustained activation in the prefrontal cortex during working memory processes (Funahashi et al., 1993), could bridge the temporal gap between the CS and the US, while O'Reilly et al. (2007) couple their model of DA activity with a neuro-computational model of working memory involving the prefrontal cortex and the basal ganglia in order to address trace conditioning (O'Reilly and Frank, 2006).

In the experiments shown in this article, the CS is an individual visual stimulus that activates specific clusters of cells in the inferotemporal cortex (IT). Object-level representations in IT allow to provide the prefrontal cortex, the amygdala and the basal ganglia with rich detailed representations of visual objects (Tanaka, 2000). However, inputs to the model could be easily adapted to auditory inputs. The US is a food reward, activating the lateral hypothalamus (LH). Neurons in LH are activated by the specific taste components of a single reward, proportionally to their magnitude (Nakamura and Ono, 1986). Rewards are therefore represented by a combination of tastes [for example fat, sugar, salt, umami, as in the MOTIVATOR model of Dranias et al., 2008) allowing to distinguish different rewards from each other by their nature instead of only their relative magnitude.

#### 2.1.2. Role of VTA and forebrain structures

The midbrain dopaminergic system is predominantly composed of the SNc and VTA. VTA plays a specific role in the facilitation of approach behaviors and incentive learning (Fields et al., 2007), while SNc is more involved in motor and cognitive processes, although this functional distinction is more based on anatomical considerations than direct observations (Haber, 2003). The proposed model focuses on VTA activation during conditioning because of its central role in the reward circuitry (Sesack and Grace, 2010), but it is not excluded that a similar behavior is observed in SNc because of the spiraling structure of striato-nigro-striatal pathways (Haber et al., 2000).

Dopaminergic neurons in VTA exhibit a relatively low tonic activity (around 5 Hz), but react phasically with a short-latency (<100 ms), short-duration (<200 ms) burst of high activity in response to unpredicted rewards, aversive, salient or novel stimuli (Schultz et al., 1993; Mirenowicz and Schultz, 1994; Horvitz, 2000; Redgrave et al., 2008). After appetitive conditioning, the same cells also react phasically to reward-predicting stimuli (Schultz et al., 1997). These phasic bursts of activity for both unpredicted rewards and reward-predicting cues are dependent on glutamatergic activation by PPTN (Dormont et al., 1998; Lokwan et al., 1999; Pan and Hyland, 2005), which is itself driven by inputs from LH and the central nucleus of the amygdala (CE) (Semba and Fibiger, 1992). Excitatory inputs from the prefrontal cortex (PFC) to VTA, PPTN and LH exert a regulatory role on this bursting behavior (Fields et al., 2007; Geisler and Wise, 2008) and regulate plasticity in VTA (Wolf et al., 2004).

The mechanisms underlying inhibitory control of VTA are less clear. VTA receives predominantly GABAergic synapses from the ventral basal ganglia (BG), especially from the ventromedial shell of the nucleus accumbens (NAcc) and the ventral pallidum (VP) (Zahm and Heimer, 1990; Usuda et al., 1998). These inhibitory projections are known to control the number of DA neurons in VTA able to switch from an hyperpolarized state to an irregular spontaneous firing rate around 5 Hz. There is also a large number of GABAergic neurons in VTA (around 30%) but they predominantly project outside VTA (Carr and Sesack, 2000). A recently labeled area posterior to the VTA, the rostromedial tegmental nucleus (RMTg), has been shown to provide a strong GABAergic inhibition on dopaminergic VTA cells, able to produce the dip observed at reward omission (Jhou et al., 2009; Lavezzi and Zahm, 2011; Bourdy and Barrot, 2012). Neurons in RMTg are excited by aversive events and reward omission, and this activation is provoked by excitatory projections from the lateral habenula (LHb) which is activated in the same conditions (Hikosaka et al., 2008; Balcita-Pedicino et al., 2011; Bromberg-Martin and Hikosaka, 2011; Hong et al., 2011).

#### 2.1.3. Role of the amygdala

The amygdala is long known for its involvement in acquiring and expressing auditory fear conditioning (LeDoux, 2000). Neurons in the basolateral amygdala (BLA), the major input structure of the amygdala, learn to associate CS and US representation, based either on thalamic or cortical information (Doyère et al., 2003), with long-term potentiation being modulated by dopaminergic innervation from VTA (Bissière et al., 2003). The output structure of the amygdala, the central nucleus of the amygdala (CE) is critical for expressing fear conditioning (conditioned responses), through its projections on various brainstem nuclei (Koo et al., 2004).

However, the amygdala is now recognized to be also involved in appetitive conditioning and reward processing (Baxter and Murray, 2002; Murray, 2007). The amygdala and LH both react to the palability of rewards, suggesting either common afferences in the brainstem, a direct projection from LH to BLA (Sah et al., 2003) or an indirect one through the gustatory thalamus, as lesions of the gustatory brainstem nuclei abolish food-elicited responses in both LH and the amygdala (Nishijo et al., 2000). In this model, we assume a direct projection from LH to BLA, but how the amygdala gets access to the value of a food reward is still not clear.

BLA neurons have been shown to respond proportionally to reward magnitude (Bermudez and Schultz, 2010). They also respond to both reward-predicting cues and the associated rewards, with a sustained activation during the delay (Ono et al., 1995; Nishijo et al., 2008). This places the BLA at a central position for learning CS-US associations, or more precisely associating the value of the US to the sensory representation of the CS. This information is transferred to CE, which is able to activate VTA, either through direct projections (Fudge and Haber, 2000)—although they are quite weak and have only been observed in primates—, or more likely indirectly through excitation of PPTN (Semba and Fibiger, 1992; Lee et al., 2011).

#### 2.1.4. Role of the ventral basal ganglia

The ventral BG plays a critical role in learning goal-oriented behaviors and is considered as an interface between the limbic and motor systems, as it receives converging inputs from the amygdala, hippocampus and prefrontal cortex (Nicola, 2007; Humphries and Prescott, 2010). Its major input structure, the ventral striatum, is mostly composed of the nucleus accumbens (NAcc), itself decomposed into core and shell territories, but also extends without a clear demarkation into the caudate nucleus and the putamen, accounting for around 20% of the whole striatum (Haber and Knutson, 2010). It is primarily composed of GABAergic medium-spiny projection neurons (MSN, 90%), as well as tonically-active cholinergic neurons (TAN) and GABAergic interneurons. MSN neurons project on the ventral pallidum (VP), VTA, SNc, LH, and PPTN. They receive inputs from VP, VTA, LH, BLA and the subiculum (part of the hippocampal formation) (Humphries and Prescott, 2010; Sesack and Grace, 2010).

NAcc is involved in learning the incentive motivational value of rewards (Robbins and Everitt, 1996; Nicola, 2007; Galtress and Kirkpatrick, 2010). Excitatory inputs from the BLA have been shown necessary to promote reward-seeking behaviors and enable the cue-evoked excitation of NAcc during operant conditioning. NAcc is also involved in Pavlovian reward learning, with single neurons being phasically activated by both CS and US after sufficient training (Day and Carelli, 2007). Learning in NAcc has been shown to depend strongly on dopaminergic innervation from VTA (Eyny and Horvitz, 2003).

VP, the output structure of the ventral BG, is also strongly involved in reward processing and reward expectation (Smith et al., 2009; Tachibana and Hikosaka, 2012). It receives GABAergic projections from NAcc, excitatory projections from PPTN, and projects to SNc/VTA, LHb, RMTg, and the mediodorsal nucleus of the thalamus (MD) (Hallanger and Wainer, 1988; Jhou et al., 2009; Haber and Knutson, 2010). During classical conditioning, VP cells are excited by reward-predicting cues and the associated reward when the reward is large, but inhibited by small rewards (Tindell et al., 2004). The NAcc → VP pathway is therefore considered a major route for disinhibiting efferent structures at CS onset and reward delivery and guide reward-orienting behaviors (Sesack and Grace, 2010).

Regarding the involvement of the ventral BG in timing, the current evidence is rather controversial. Two lesion studies showed no involvement of NAcc in the timing of instrumental responding (Meck, 2006; Galtress and Kirkpatrick, 2010), but Singh et al. (2011) showed that lesions of NAcc induce a deficit in learning the timing of Pavlovian responses. The NAcc and the medial caudate nucleus robustly activate during reward anticipation (Deadwyler et al., 2004), while the rostroventral putamen most reliably deactivates in response to non-reward delivery (McClure et al., 2003; O'Doherty et al., 2003). Lesions of NAcc have recently been shown to disrupt reinforcement-omission effects (Judice-Daher and Bueno, 2013). However, no cellular recordings have yet shown that NAcc cells react specifically to reward omission.

In this model, we form the hypothesis that a subset of NAcc cells learns the precise time when a reward is expected and gets activated when it is omitted. Recent advances in the neurobiology of interval timing show that a similar mechanism is likely to occur in the dorsal striatum during peak-interval tasks (Matell and Meck, 2004; Coull et al., 2011). The *Striatal-Beat Frequency* model (Matell and Meck, 2000; Lustig et al., 2005) has proposed that striatal cells act as coincidence detectors, learning to react to a particular configuration of cortical inputs when a DA burst occurs and to signal the temporal expectation of reward. In this framework, cortical inputs oscillate at various frequencies in the alpha range (8–13 Hz) and are synchronized at cue-onset. This provides an unique population code for the time elapsed since cue onset, so striatal cells can learn to react to a specific duration through dopamine-modulated long-term potentiation (LTP) or depression (LTD) (Calabresi et al., 2007; Shen et al., 2008). We consider a similar mechanism here for learning CS-US interval durations in NAcc.

Synaptic plasticity at corticostriatal synapses depends on the polarization of the membrane potential: in the hyperpolarized state (−90 mV, called the down-state), striatal cells exhibit mostly LTD at active synapses; in the depolarized state (−60 mV, the up-state), these cells exhibit LTP or LTD depending on the extracellular dopamine level (Calabresi et al., 2007; Shen et al., 2008). Neurons in NAcc exhibit these up- and down-states (O'Donnell and Grace, 1995), and the transition from the down-state to the up-state depends either on phasic DA release from VTA (Gruber et al., 2003; Goto and Grace, 2005), afferent input from the ventral subiculum of the hippocampus (O'Donnell and Grace, 1995) or a conjunction of medial prefrontal cortex and amygdala inputs (McGinty and Grace, 2009). This mechanism is thought to help restricting striatal firing to the exact time when reward is expected: NAcc cells are brought in the up-state by DA bursts at reward delivery, allowing the to learn the precise cortical pattern. After learning the same cell could be brought in the up-state only by this cortical pattern (in conjunction with BLA inputs), even if VTA is not bursting (Matell and Meck, 2004).

### 2.2. The proposed model

#### 2.2.1. Overview

**Figure 1:**
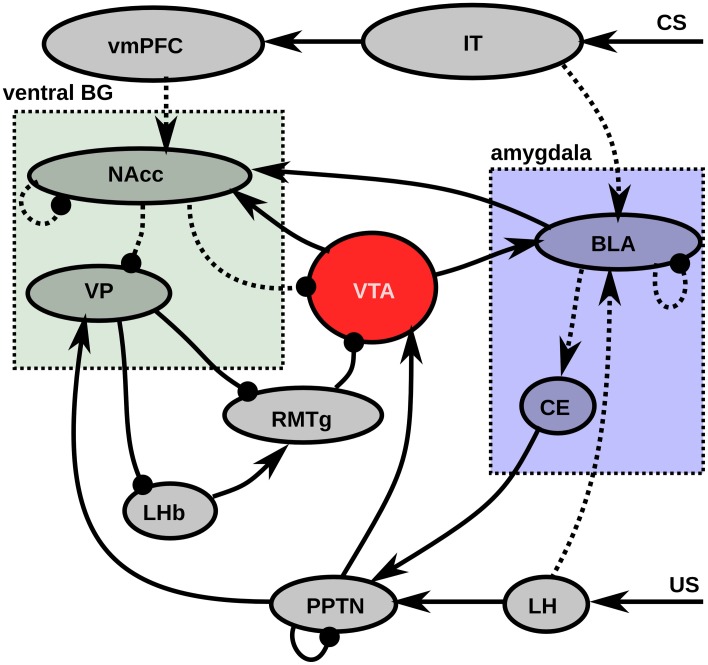
**Functional description of the model**. Pointed arrows represent excitatory connections, rounded arrows represent inhibitory projections. Dashed lines represent learnable connections, while solid represent fixed connections. LH signals US delivery to BLA (Sah et al., 2003) and PPTN (Semba and Fibiger, 1992). IT encode a visual representation of the CS, which activates BLA (Cheng et al., 1997) and vmPFC (Carmichael and Price, 1995). BLA learns to associates the CS and US representations under the modulatory influence of the DA released by VTA (Bissière et al., 2003) and projects on CE (LeDoux, 2000) which excites PPTN (Semba and Fibiger, 1992). The excitatory projection from PPTN to VTA is able to provoke phasic DA bursts (Lokwan et al., 1999). NAcc MSN neurons receives excitatory projections from BLA (Ambroggi et al., 2008) and vmPFC (Haber, 2003) and learning is modulated by DA release from VTA (Robbins and Everitt, 1996). They inhibit VTA dopaminergic neurons (Usuda et al., 1998) and VP (Zahm and Heimer, 1990). VP also receives excitatory projections from PPTN (Hallanger and Wainer, 1988) and inhibits both LHb and RMTg (Haber and Knutson, 2010). LHb excites RMTg (Balcita-Pedicino et al., 2011) which in turn inhibits VTA (Jhou et al., 2009). Abbreviations: LH, lateral hypothalamus; IT, inferotemporal cortex; BLA, basolateral nucleus of the amygdala; CE, central nucleus of the amygdala; vmPFC, ventromedial prefrontal cortex; PPTN, pedunculopontine nucleus; VTA, ventral tegmental area; NAcc, nucleus accumbens; VP, ventral pallidum; LHb, lateral habenula; RMTg, rostromedial tegmental nucleus.

In this section, we will explain the major flows of information and learning in the model before describing more precisely the details of the model, depicted on Figure 1. Most experiments in this article will concern the concurrent learning of three different CS-US associations, each using different visual and gustatory representations, and with different CS-US intervals (see section 2.2.3). The first phase of learning represents sensitization to the rewards, by presenting each reward individually ten times. The US representation activates a set of cells in LH, depending of the basic tastes composing it, what in turn activates the US-selective population of PPTN, provoking a phasic DA burst in VTA which gates learning in BLA. After sufficient exposure to each reward, BLA has self-organized to represent them individually by the activation of a single cell. Meanwhile, BLA progressively learns to activate CE, which in turn activates the CS-selective population of PPTN (Figure 1). However, when reward is delivered, the preceding activation of the US-selective population inhibits activation in the CS-selective one. During the sensitization phase, a similar self-organizatory mechanism occurs in NAcc: individual rewards become represented by different single neurons.

The second phase of learning concerns conditioning *per se* with distinct trials for each CS-US association: an initially neutral visual stimulus (CS) activates a distributed representation in IT, which lasts for a fixed duration before the US is delivered. This visual representation projects onto BLA, and, through DA-modulated learning in BLA at reward-delivery, becomes able through repetitive pairing to activate the same BLA cell that would be activated by the US alone. Homeostatic regulation in BLA ensures that the BLA activity at CS onset has the same amplitude as the reward-related activity. CS-related activation in BLA becomes able to activate CE, which becomes able to provoke VTA bursts through excitation of PPTN. This mechanism is sufficient to explain the progressive phasic DA bursts in VTA at CS onset during learning.

In parallel, CS onset activates a bank of oscillators in the ventromedial prefrontal cortex (vmPFC) at different frequencies. During conditioning, the phasic DA burst at US delivery brings the corresponding NAcc cell into the up-state, allowing it to become selective to the precise configuration of cortical oscillators corresponding to the elapsed duration since CS onset. This progressive activation at US delivery diminishes the amplitude of the US-related VTA burst through the direct NAcc → VTA inhibitory projection. Meanwhile, NAcc learns to inhibit VP at reward delivery, what could potentially lead to the disinhibition of LHb, provoking a dip of activity in VTA through RMTg. However, reward delivery activates the US-selective population of PPTN, which excites VP: the inhibitory influence of NAcc is counterbalanced by PPTN, what leaves VP above its baseline level and avoid unwanted inhibition of VTA.

After a sufficient number of conditioning trials, we investigate reward omission, where the CS is presented for the usual duration, but not the US. In this case, one NAcc cell goes into the up-state when the reward is expected because of its strong vmPFC input at this time and inhibits VP. This inhibition is then not counterbalanced anymore by US-related PPTN activation, so this disinhibits LHb, activates RMTg and finally provokes a strong inhibition of VTA, bringing it below baseline for a certain duration (the dip).

#### 2.2.2. Computational principles

Each area in the proposed model is composed of a given number of computational units, where each unit computes the mean activity of a population of neurons. The dynamics of each unit is described by the evolution of its time-dependent firing rate (Dayan and Abbott, 2001). The firing rate *r*(*t*) of an unit is a positive scalar describing the instantaneous number of spikes per second emitted by neurons in the corresponding population. In this model, it is taken to be the positive part of the so-called *membrane potential m*(*t*) of the unit, which follows a first order differential equation depending on the firing rate of other units. In this model, the absolute value of the firing rate is usually restricted to the range [0, 1] through homeostatic regulation of learning (see for example the Equation 12), where 1 represents the maximal instantaneous firing rate that the considered type of cell can have. Typical units in the model are governed by Equations (1, 2):

(1) $$\tau \cdot \frac{dm(t)}{dt}+m(t)=g_{\text{exc}}(t)-g_{\text{inh}}(t)+B+\eta (t)$$

(2) $$\;r(t)={\left(m(t)\right)}^{+}$$

where τ is the time constant of the cell (expressed in milliseconds), *B* is its baseline activity, η(*t*) an additive noise term chosen randomly at each time step from an uniform distribution between −0.1 and 0.1, *g*_exc_(*t*) and *g*_inh_(*t*) being the weighted sum of excitatory and inhibitory afferent firing rates, respectively. ()^+^ is the positive function, which only keeps the positive part of the operand and outputs 0 when it is negative. In the rest of this article, we will only describe how the membrane potential *m*(*t*) of each unit evolves, the corresponding firing rate being always the positive part.

Units in this model can differentially integrate their inputs depending on their assigned type (here exc, inh, mod and dopa). This type corresponds either to the neurotransmitter type (exc and mod represent glutamergic synapses, inh GABAergic ones and dopa represents dopaminergic receptors) or the region of origin (exc and mod connections have both an excitatory effect but arise from different areas and are integrated differently).

For a given type of synapses, the weighted sum of inputs is defined by Equation (3):

(3) $$g_{\text{type}}(t)=\sum_{i}^{\text{type}}w_{i}(t)\cdot r_{i}(t)$$

where *i* is the index of a synapse of this type, *r*_*i*_(*t*) the firing rate of the presynaptic neuron at time *t* and *w*_*i*_(*t*) the weight of the connection (or synaptic efficiency).

Some computational principles in this model rely on the conversion of the onset of a tonic input *x*(*t*) (reward delivery, CS presentation) into a short-term phasic component. For convenience, we define here a function Φ_τ, *K*_(*x*) allowing this transformation according to Equations (4, 5):

(4) $$\tau \cdot \frac{d\bar{x}(t)}{dt}+\bar{x}(t)=x(t)$$

(5) $$\;\Phi_{\tau ,k}(x(t))={\left(x(t)-k\cdot \bar{x}(t)\right)}^{+}$$

**Figure 2:**
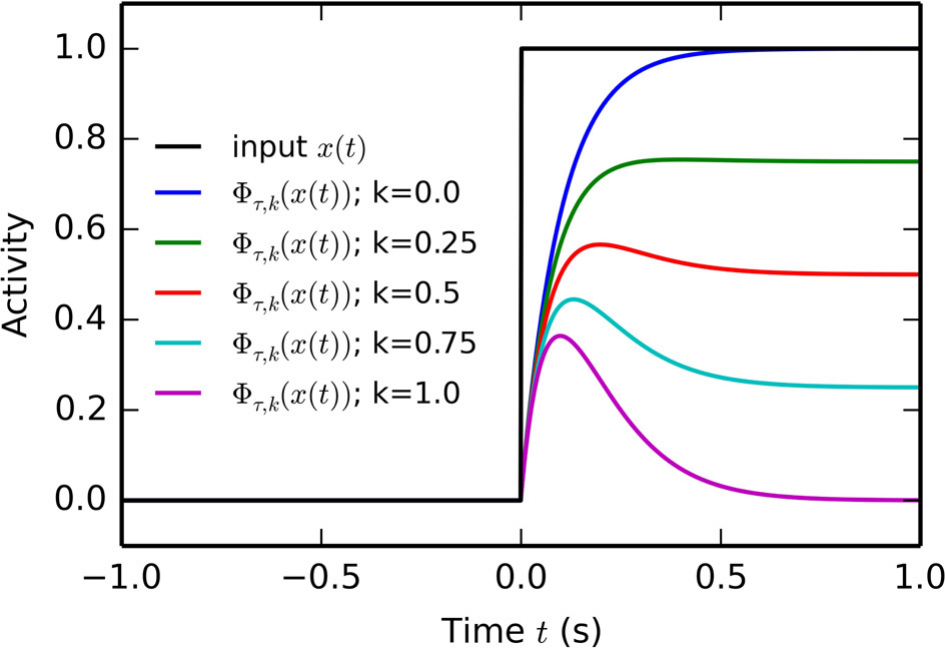
**Temporal profile of the phasic function Φ_τ, *k*_(*x*) defined by Equation (5)**. At *t* = 0, the Heaviside input *x*(*t*) goes from 0 to 1. The temporal profile of five phasic functions Φ_τ, *k*_(*x*) with τ = 50 ms and *k* ranging from 0 to 1 is displayed. If *k* = 0, the phasifunction is a simple leaky integrator with time constant τ. If *k* = 1, the output of the filter is a localized bump peaking at *t* = τ and converging toward 0.

*x*(*t*) integrates the input *x*(*t*) with a time constant τ, while Φ_τ_(*x*(*t*)) represents the positive part of the difference between *x*(*t*) and *x*(*t*). *k* is a parameter controlling which proportion of the input will be kept on the long-term (if *k* = 0 the tonic component is preserved, if *k* = 1 ϕ_τ, *k*_(*x*(*t*)) will converge toward zero). If *x*(*t*) is for example an Heaviside function (switching from 0 to 1 at *t* = 0), Φ_τ, 0_(*x*(*t*)) will display a localized bump of activation with a maximum at *t* = τ, as depicted on Figure 2.

Another useful function is the threshold function, which outputs 1 when the input exceeds a threshold Γ, 0 otherwise (Equation 6):

(6) $$\Delta_{\Gamma }(x)=\{\begin{array}{l} 0\text{ if }x<\Gamma \\ 1\text{ otherwise.} \end{array}$$

The learning rules used in the model derive from the Hebbian learning rule. The simplest variant of this learning rule in the model is a thresholded version described in Equation (7). The evolution over time of the weight *w*_*i*,*j*_(*t*) of a synapse between the neuron *i* in population pre (presynaptic neuron) and the neuron *j* of population post (postsynaptic neuron) is governed by:

(7) $$\epsilon \cdot \frac{dw_{i,j}(t)}{dt}={\left(r_{\text{pre}}^{i}(t)-\theta_{\text{pre}}\right)}^{+}\cdot {\left(r_{\text{post}}^{j}(t)-\theta_{\text{post}}\right)}^{+}$$

where *r*^*i*^_pre_(*t*) and *r*^*j*^_post_(*t*) are the pre- and post-synaptic firing rates, θ_pre_ and θ_post_ are fixed thresholds, and ϵ is the learning rate. The thresholds can be adjusted to take baseline firing rates into account and restrict learning to significant deviations from this baseline. Weight values are restricted to the range [*w*_min_, *w*_max_], where *w*_min_ is usually 0.

Another learning rule used in the model derives from the covariance learning rule (Dayan and Abbott, 2001; Vitay and Hamker, 2010; Schroll et al., 2012). In this framework, only those cells whose firing rate is significantly above the mean firing rate in their respective population can participate to learning. The evolution over time of the weights is described by the Equation (8):

(8) $$\epsilon \cdot \frac{dw_{i,j}(t)}{dt}={\left(r_{\text{pre}}^{i}(t)-{\bar{r}}_{\text{pre}}(t)\right)}^{+}\cdot {\left(r_{\text{post}}^{j}(t)-{\bar{r}}_{\text{post}}(t)\right)}^{+}$$

where *r*_pre_(*t*) and *r*_post_(*t*) are the average firing rate in the pre- and post-synaptic populations, respectively. This mean activity allows to adapt more dynamically the learning behavior between two populations. Dopamine-modulated learning rules will be described in the rest of the text, together with the corresponding populations (BLA and NAcc). The parameters of all learning rules are described in Table 2.

All equations in the model are solved using the forward Euler method, with a time step of 1 ms. The model is implemented in the neurosimulator ANNarchy^1^ (Artificial Neural Network architect), which combines a Python interface to a high-performance parallel simulation kernel in C++.

#### 2.2.3. Representation of inputs

The network is presented with two kinds of inputs: the visual representation of the CS and the gustatory representation of the US. In this article, we will concurrently learn three CS-US associations (CS1 + US1, CS2 + US2, CS3 + US3), with different parameters (magnitude and time interval) in order to show the robustness of the model. Other combinations of magnitude and duration provoke similar results of the model.

The CS are represented by a three-dimensional binary vector, where each element represents the presence (resp. absence) of the corresponding CS with a value of 1 (resp. 0). The US are represented by a four-dimensional vector, where each element represents a single taste component (for example salt, sugar, fat and umami as in Dranias et al., 2008). As shown in Table 1, there is an overlap between the different tastes of the US, rendering harder the task to distinguish them. Moreover, each US representation is multiplied by a magnitude, representing the quantity of food delivered. In this article, this magnitude is the same for all tastes composing the US.

**Table 1:** **Definition of the inputs to the model**.

**Number**	**CS**	**US**	**Magnitude**	**Interval (s)**
1	[1, 0, 0]	[1, 1, 0, 0]	0.8	2
2	[0, 1, 0]	[1, 0, 1, 0]	0.5	3
3	[0, 0, 1]	[1, 0, 1, 1]	1.0	4

Each CS-US association is defined by unique CS and US vectors. During conditioning, rewards are presented with a certain magnitude, and after a certain delay after CS onset.

A conditioning trial is composed of a first reset interval of 1 s where no input is given to the network (all elements of the CS and US representations are set to 0). At time *t* = 1s, the CS representation is set to the corresponding vector. This input is maintained for a given duration, whose value depend on the CS-US association (2 s for CS1-US1, 3 s for CS2-US2, 4 for CS3-US3). These different interval durations are chosen to show that the network can indeed learn different CS-US intervals without any modification, but different combinations would lead to similar results.

Once the delay is elapsed, the US representation is set for 1 s, with the CS representation maintained. In extinction trials, the US representation is not set. After this duration of 1 s, all elements of the CS and US representations are reset to 0, and the network can settle for one more second, so the duration of one trial is equal to the interval plus 3 s.

The visual input to the model is represented by the population IT, composed of nine units. The CS representations activate different neurons in IT with a specific one-to-many pattern: one element of the CS vector activates exactly three units in IT (called a cluster), without overlap. This activation is excitatory, with a fixed weight value of 1.0 (see Table 2 for the weight value of all projections.). Each neuron in IT has a membrane potential governed by Equation (9), with the firing rate being its positive part (Equation 2):

(9) $$\tau \cdot \frac{dm(t)}{dt}+m(t)=g_{\text{exc}}(t)+\eta (t)$$

with τ = 10 ms, η(*t*) randomly chosen at each time step in [−0.1, 0.1] and *g*_exc_(*t*) the input from the CS representation. The gustatory inputs are similarly represented by LH, with a one-to-one projection (one neuron in LH represents one element of the US representation). Thus, neurons in LH are also governed by Equation (9), with τ = 10 ms.

**Table 2:** **Parameters of the projections in the model**.

**Pre**	**Post**	**Type**	**Pattern**	**Eq**.	**Weight**	**[*w*_min_, *w*_max_]**	**ϵ**	**θ_pre_**	**θ_post_**	***K***	**τ_dopa_**	***k***	**τ_α_**
VIS	IT	Exc	One-to-many	–	1.0	–	–	–	–	–	–	–	–
GUS	LH	Exc	One-to-one	–	1.0	–	–	–	–	–	–	–	–
LH	BLA	Exc	All-to-all	11	0.3 ± 0.2	[0, −]	100	–	–	10	100	1	1
IT	BLA	Mod	All-to-all	13	0.0	–	300	–	–	–	–	–	–
BLA	BLA	Inh	All-to-all	8	0.5	[0, 3]	100	–	–	–	–	–	–
BLA	CE	Exc	All-to-all	–	1.0	–	–	–	–	–	–	–	–
CE	PPTN	Exc	All-to-one	–	1.5	–	–	–	–	–	–	–	–
LH	PPTN	Exc	All-to-one	–	0.75	–	–	–	–	–	–	–	–
PPTN	PPTN	Inh	All-to-all	–	2	–	–	–	–	–	–	–	–
PPTN	VTA	Exc	All-to-all	–	1.5	–	–	–	–	–	–	–	–
PPTN	VP	Exc	All-to-all	–	0.5	–	–	–	–	–	–	–	–
VP	RMTg	Inh	All-to-all	–	1	–	–	–	–	–	–	–	–
VP	LHb	Inh	All-to-all	–	3	–	–	–	–	–	–	–	–
LHb	RMTg	Exc	All-to-all	–	1.5	–	–	–	–	–	–	–	–
RMTg	VTA	Inh	All-to-all	–	1.0	–	–	–	–	–	–	–	–
IT	vmPFC	Exc	Many-to-many	–	0.3	–	–	–	–	–	–	–	–
vmPFC	NAcc	Mod	All-to-all	11	0	[−0.2, −]	50	–	–	5	10	1	10
BLA	NAcc	Exc	One-to-one	–	0.3	–	–	–	–	–	–	–	–
VTA	NAcc	Dopa	All-to-all	–	0.5	–	–	–	–	–	–	–	–
NAcc	NAcc	Inh	All-to-all	8	0.5	[0, 1]	1000	–	–	–	–	–	–
NAcc	VP	Inh	All-to-all	7	0	[0, 2]	100	0	0.5	–	–	–	–
NAcc	VTA	Inh	All-to-all	7	0	[0, 2]	500	0	0	–	–	–	–

*Pre and Post describe the pre- and post-synaptic populations, respectively. Type denotes the type of the synapses in the projection, as they are differentially integrated by the postsynaptic neurons (exc, inh, mod, dopa). Pattern denotes the projection pattern between the pre- and post-synaptic populations: all-to-all means that all post-synaptic neurons receive connections from all presynaptic neurons; one-to-one means that each postsynaptic neuron receives exactly one connection from the pre-synaptic population, without overlap. one-to-many and many-to-many refer to specific projection patterns for the clusters in IT, please refer to section 2.2.3 for a description. Eq represents the number of the equation governing plasticity in the projection. Weight describe the initial value for the weight of each synapse (non-learnable connections keep this value through the simulation). *w*_min_ is the minimal value that a learnable weight can take during learning, while *w*_max_ is the maximal value (if any). The other parameters correspond to the respective equations of the learning rules, please refer to them for details*.

#### 2.2.4. Amygdala

The amygdala is decomposed into its input structure, BLA, and its output structure, CE. BLA receives visual information from IT, gustatory information from LH and dopaminergic innervation from VTA. Its role is to learn to associate the CS and US representations: a BLA cell which was previously activated by the food reward alone, proportionally to its magnitude (Bermudez and Schultz, 2010), should become activated with the same firing rate at CS onset, indicating a transfer of the value of the US to the CS.

**Figure 3:**
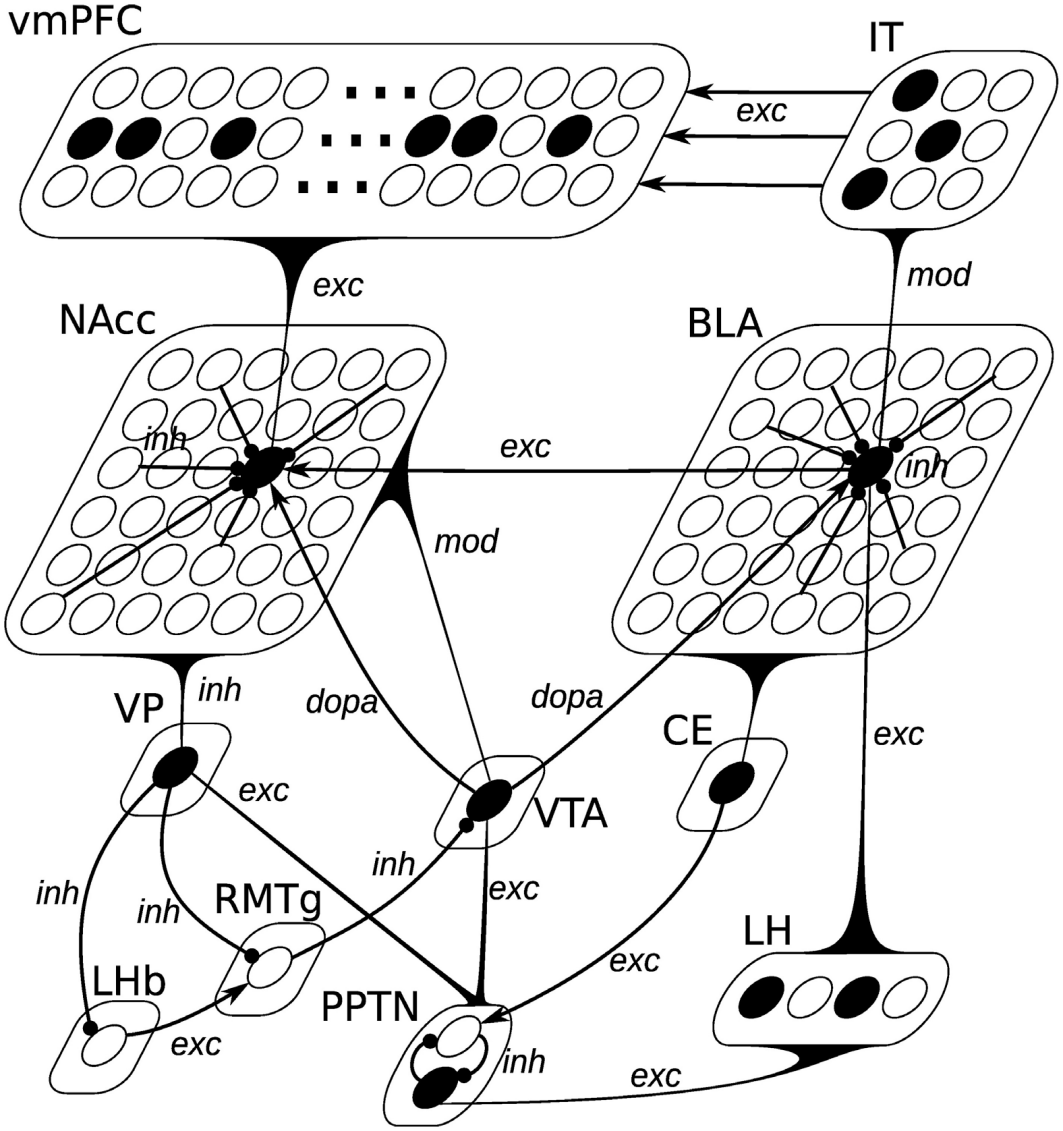
**Neural network description of the model**. Pointed arrows represent excitatory or dopaminergic synapses, while rounded arrows represent inhibitory synapses. The black curved triangles represent connections from all units of a given population to a single cell. The type of the connection (exc, mod, inh, dopa) is added next to the arrow. Lateral inhibitory connections within BLA and NAcc are only partially represented for simplicity. BLA is composed of 36 units, whose activation is defined by Equation (10). Each unit receives excitatory connections from all LH units (*g*_exc_(*t*)), modulated connections from all IT units (*g*_mod_(*t*)), one dopaminergic connection from VTA (*g*_dopa_(*t*)) and inhibitory connections from all other BLA units (*g*_inh_(*t*)). Each of the 3 banks of 50 oscillators in vmPFC receives excitatory connections (*g*_exc_(*t*)) from a specific cluster of three units in IT representing a given CS. NAcc is composed of 36 units, whose activation is defined by Equation (16). Each unit receives a single excitatory connection from BLA (*g*_exc_(*t*)), excitatory connections from all units of vmPFC (*g*_mod_(*t*)), one dopaminergic connection from VTA (*g*_dopa_(*t*)) and inhibitory connections from all other NAcc units (*g*_inh_(*t*)). The other populations are composed of single units, integrating excitatory or inhibitory inputs.

As depicted on Figure 3, the BLA is composed of 36 units, reciprocally connected with each other through inhibitory connections (inh). Excitatory connections from LH (exc) interact with the excitatory ones from IT (labeled as mod): when no LH activation is present, a neuron can be activated solely by its excitatory inputs from IT; when LH is activated, inputs from IT do not drive the cell response. Such a non-linear interaction between different inputs may be mediated through the somatostatin-containing interneurons in BLA, which are able to suppress excitatory inputs to pyramidal cell distal dendrites (presumably from the cortex), but let them react to the inputs from LH (Muller et al., 2007). A BLA unit in this model therefore averages the behavior of pyramidal excitatory neurons, somatostatin- and parvalbumin-containing inhibitory interneurons into a single equation.

The membrane potential of each cell is driven by the Equation (10):

(10) $$\begin{array}{l} \tau \cdot \frac{dm(t)}{dt}+m(t)=\Phi_{\tau_{\text{exc}},k}\left(g_{\text{exc}}(t)\right)+\left(1-\Delta_{\Gamma }\left(g_{\text{exc}}(t)\right)\right)\cdot \\ \;\Phi_{\tau_{\text{mod}},k}\left(g_{\text{mod}}(t)\right)-g_{\text{inh}}(t)+\eta (t) \end{array}$$

where τ = 10 ms is the time constant of the cell, τ_exc_ = τ_mod_ = 500 ms are the integration constants for the phasic functions of inputs, *k* = 0.8 is a parameter ensuring that the cell still responds with a significant firing rate after the phasic component is processed, Γ = 0.1 is a threshold on the excitatory inputs ensuring that modulated inputs from IT can only drive the cell's activity when the input from LH is absent. The effect of this complex equation will be explained with more details in section 3.1.

CE is composed of a single unit, receiving excitatory inputs from all BLA units. Its membrane potential is driven by the Equation (9), with τ = 10 ms. As only one unit is active at a time in BLA because of lateral inhibition, CE simply copies activity in BLA, regardless the CS-US association.

Learning occurs in BLA for three types of connections: the excitatory input from LH, the modulated input from IT and the inhibitory lateral connections between the BLA neurons. The learning procedure is composed of two phases: in the sensitization phase, the US are presented alone, without any CS. This allows BLA to learn to represent each US by a single neuron. In the conditioning phase, learning in the LH → BLA pathway is reduced. This represents the fact that the formation of food reward representations in BLA is a much slower process than the conditioning sessions.

Excitatory connections from LH to BLA are learned with a dopamine-modulated covariance-based learning, with the addition of a homeostatic mechanism to ensure the weights do not increase infinitely. The evolution of these weights is described by Equation (11):

(11) $$\begin{array}{l} \epsilon \cdot \frac{dw_{i,j}(t)}{dt}=K\cdot \Phi_{\tau_{\text{dopa}},k}\left(g_{\text{DA}}(t)\right)\cdot \text{OR}(r_{\text{pre}}^{i}(t)-{\bar{r}}_{\text{pre}}(t),\\ \;r_{\text{post}}^{j}(t)-{\bar{r}}_{\text{post}}(t))-\alpha^{j}(t)\cdot r_{\text{post}}^{j}{\left(t\right)}^{2}\cdot w_{i,j}(t) \end{array}$$

with ϵ = 100 in the sensitization phase and 10,000 in the conditioning phase, *K* = 10, τ_dopa_ = 100 ms, *k* = 1. In the first term of the equation, the covariance term is modulated by a value depending on the dopaminergic activity in VTA. This allows DA extracellular levels to influence the induction of LTP in BLA, as experimentally observed (Bissière et al., 2003). It is filtered through the phasic function Φ_τ_DA_, *k*_ (*g*_dopa_(*t*)) with *k* = 1, so that DA-mediated learning only takes temporarily place when DA is significantly above its baseline, i.e., during a phasic burst of activation.

This first term also differs from the covariance learning rule described by Equation (8), as it uses a OR(*x*, *y*) function, being OR(*x*, *y*) = *x* · *y* if *x* > 0 or *y* > 0 and OR(*x*, *y*) = 0 if both *x* < 0 and *y* < 0. If both cells are significantly more activated than their respective population, the term is positive and LTP is engaged. If only one cell is significantly active (either pre- or post-synaptic), the term is negative and LTD appears (homo- or hetero-synaptic LTD, respectively). This simple behavior allows to develop a high selectivity for specific patterns in the presynaptic population. In the case where both cells are inactive (*r*^*i*^_pre_(*t*) < *r*_pre_(*t*) and *r*^*j*^_post_(*t*) < *r*_post_(*t*)), the covariance term would be positive but we set it artificially to 0, in order to avoid that silent neurons build up strong connections.

The second term of the learning rule implements a regularization term derived from the Oja learning rule (Oja, 1982) ensuring that the postsynaptic activity does not increase indefinitely during learning (Vitay and Hamker, 2010; Schroll et al., 2012). This mechanism implements homeostatic plasticity whose role is to keep neurons in an energetically efficient mode (Turrigiano, 2008). As formulated in Equation (12), the regularization term α(*t*) becomes positive whenever the postsynaptic neuron fires above a certain threshold, thereby down-scaling the most active connections to this neuron:

(12) $$\tau_{\alpha }\frac{d\alpha^{j}(t)}{dt}+\alpha^{j}(t)={\left(r_{\text{post}}^{j}(t)-r_{\text{max}}\right)}^{+}$$

*r*_max_ = 1 being the postsynaptic firing rate above which regularization is engaged.

The modulated projection from IT to BLA follows a different learning rule: its principle is that this projection should learn to activate a BLA neuron with the same strength as the corresponding US. Learning is also modulated by dopamine release, as described by the Equation (13):

(13) $$\begin{array}{l} \epsilon \cdot \frac{dw_{i,j}(t)}{dt}=\Delta_{\Gamma_{\text{dopa}}}\left(g_{\text{dopa}}^{j}(t)\right)\cdot \left(r_{\text{pre}}^{i}(t)-{\bar{r}}_{\text{pre}}(t)\right)\cdot \\ \;\left(r_{\text{post}}^{j}(t)-{\bar{r}}_{\text{post}}(t)\right)\cdot {\left(g_{\text{exc}}^{j}(t)-g_{\text{mod}}^{j}(t)\right)}^{+} \end{array}$$

with Γ_dopa_ = 0.3 being a threshold on VTA activity. The term (*g*_exc_(*t*) − *g*_mod_(*t*))^+^ ensures that the modulated projections stop learning whenever their net effect on a postsynaptic neuron exceeds the one of the excitatory projection from LH during DA bursts.

Lateral inhibitory connections between BLA cells are learned according to the covariance-based learning rule described in the Equation (8), forcing competition between the cells and ensuring that only one BLA cell is active for a single stimulus.

#### 2.2.5. Pedunculopontine nucleus

PPTN is involved in generating phasic DA bursts in VTA for both reward-predicting cues and rewards through direct glutamatergic projections (Pan and Hyland, 2005). Two different populations of PPTN neurons signal CS- and US-related signals to VTA (Kobayashi and Okada, 2007). In the model, PPTN is therefore composed of two units, one receiving US information from LH, the other CS information from CE, as depicted on Figure 3. These two neurons are moreover inhibiting each other, so that only one is active at a given time. The dynamics of these neurons are described by the same Equation (14), the only difference being the origin of the excitatory information:

(14) $$\tau \cdot \frac{dm(t)}{dt}+m(t)=\Phi_{\tau_{\text{exc}},k}\left(g_{\text{exc}}(t)\right)-g_{\text{inh}}(t)+\eta (t)$$

with τ = 10 ms, τ_exc_ = 50 ms, and *k* = 1.

#### 2.2.6. Ventromedial prefrontal cortex

As in the Striatal-beat frequency model (Matell and Meck, 2004), we model the cortical inputs to NAcc by a bank of oscillators synchronized at CS onset. Each CS is represented by a group of 50 units oscillating at various frequencies between 2 and 8 Hz. Indeed, enhanced top–down synchrony in the extended theta band has been observed between vmPFC and NAcc during reward anticipation (Cohen et al., 2012).

As three CS are used in the experiments presented in this article, there are three banks of 50 units, each activated by the corresponding cluster in IT. When the sum of excitatory inputs exceeds a given threshold *T*_start_ = 0.8, the current time *t* of the simulation is stored in the variable *t*_0_, and the membrane potential of each unit varies according to the Equation (15):

(15) $$\tau \cdot \frac{dm(t)}{dt}+m(t)=\frac{1+\text{sin​}\left(2\pi \cdot f\cdot \left(t-t_{0}\right)+\varphi \right)}{2}$$

with τ = 1 ms, *f* the frequency of the oscillator randomly chosen at the beginning of the simulation in the range [2, 8] (uniform distribution) and ϕ the phase of the oscillator randomly chosen in the range [0, π]. When the excitatory input falls below a threshold *T*_stop_ = 0.2, the membrane potential is set to 0. Contrary to the rest of the network, this mechanism is not biologically plausible, but it abstracts the behavior of a coupled network of excitatory and inhibitory neurons, all activated by CS onset and interacting with different synaptic strengths and delays.

#### 2.2.7. Nucleus accumbens

As described by Figure 3, NAcc is composed of 36 units, integrating excitatory inputs from BLA with a one-to-one pattern (each NAcc neuron receives a connection from only one neuron in BLA), excitatory inputs from vmPFC (all-to-all), dopaminergic inputs from VTA and lateral inhibitory connections forcing competition between NAcc cells. Their membrane potential can be either in a hyperpolarized *down-state* or in a depolarized *up-state*, depending on several factors: (1) spontaneous transition from the down-state to the up-state have been described, exhibiting rhythmic delta-frequency (0.5–2 Hz) activities in freely moving rats (Leung and Yim, 1993); (2) Phasic DA release from VTA can bring NAcc neurons in the up-state (Gruber et al., 2003; Goto and Grace, 2005); (3) Massive input from the prefrontal cortex (together with hippocampal input, not modeled here) can also force this transition (McGinty and Grace, 2009).

Consequently, each unit of NAcc has an additional input variable *s*(*t*) describing its current state, taking the value −0.9 in the down-state and −0.4 in the up-state. Its effect is that the neuron can more easily have a non-zero firing rate in the up-state than in the down-state. The membrane potential of each NAcc cell evolves according to the Equation (16):

(16) $$\tau \cdot \text{​}\frac{dm(t)}{dt}\text{​}+\text{​}m(t)=g_{\text{exc}}(t)\text{​}-\text{​}g_{\text{inh}}(t)+g_{\text{dopa}}(t)+s(t)+\eta (t)$$

with τ = 10 ms. The corresponding firing rate is restricted to the range [0, 1.1]. Transitions between the two states are followed by another variable *s*_time_(*t*), which integrates *s*(*t*) over time, as described by the Equation (17):

(17) $$\tau \cdot \frac{ds_{\text{time}}(t)}{dt}+s_{\text{time}}(t)=s(t)$$

with τ = 450 ms. The role of the variable *s*_time_(*t*) is to ensure spontaneous transitions between the up- and down-states in the absence of external inputs or dopaminergic activation. Transitions from the down-state to the up-state are provoked by one of the following events:

- The activity of VTA exceeds a threshold Γ_dopa_ = 0.3;
- Excitatory inputs *g*_exc_(*t*) exceed the threshold Γ_glut_ = 1;
- The variable *s*_time_(*t*) exceeds the threshold Γ_up_ = −0.45.

Transitions from the up-state to the down-state are provoked by the combination of these two conditions:

- The activity of VTA is below the threshold Γ_dopa_ = 0.3;
- The variable *s*_time_(*t*) is below the threshold Γ_down_ = −0.85.

The role of the variable *s*_time_(*t*) is therefore to ensure spontaneous transitions from the down-state to the up-state, regardless other inputs. It also ensures that the NAcc cell stays long enough in the up-state before going back to the down-state when the other inputs fade away.

The mechanism proposed to exhibit up- and down-state fluctuations in our model of NAcc is a phenomenological abstraction of the underlying biological components, sufficient to reproduce some of their functional properties. A more detailed modeling approach is needed to better describe and understand the observed patterns in the context of temporal prediction. It could rely on existing biophysically-detailed models of striatal spiny neurons, studying the effects on membrane bistability of slow and fast potassium currents (Gruber et al., 2003), NMDA/AMPA receptors ratio (Wolf et al., 2005), or D1-receptor activation (Humphries et al., 2009), for example.

Excitatory inputs from vmPFC are learned using the same dopamine-modulated learning rule as the LH → BLA projection, described by the Equations (11, 12), with ϵ = 50, *K* = 5, τ_dopa_ = 10 ms, *k* = 1, τ_α_ = 10 ms, and *r*_max_ = 1. This three-factors rule covers some known effects of dopamine on corticostriatal learning (Reynolds and Wickens, 2002; Calabresi et al., 2007; Shen et al., 2008): phasic DA release potentiates learning; LTP requires both DA release, presynaptic activity and postsynaptic depolarization; strong presynaptic activation when the postsynaptic cell is in the down-state leads to LTD. The third condition of the learning rule, called heterosynaptic LTD where only the post-synaptic cell is active but not the pre-synaptic one, has not been observed in the striatum but in the hippocampus (Doyere et al., 1997). However, low-frequency stimulation at 1 Hz engage LTD at corticostriatal synapses (Fino et al., 2005), so such a mechanism can not be ruled outgnote. The known influence of dopamine depletion on corticostriatal learning is not used in this model.

τ_α_ is set very low, restricting learning to the early phase of the dopaminergic burst of VTA activity. The weights between vmPFC and NAcc are allowed to become negative (*w*_min_ = −0.2) to reflect the role of accumbal interneurons (TANs and GABAergic) in timing processes (Apicella et al., 2009; Coull et al., 2011). This particularity is essential for the adequate temporal response of NAcc neurons. Inhibitory lateral connections between NAcc cells are learned according to the covariance-based learning rule described by the Equation (8).

#### 2.2.8. Ventral pallidum

During classical conditioning, VP cells are excited by large rewards and the cues predicting them, but are inhibited by small rewards (Tindell et al., 2004). While the major source of inhibition is clearly NAcc, the source of excitation is still unknown. Based on known anatomical connections, we hypothesize that this phasic excitation is transmitted by PPTN (Hallanger and Wainer, 1988). However, when a reward is fully predicted and delivered, NAcc is activated and cancels the excitation provided by PPTN. We propose a mechanism where VP is inhibited by NAcc activation unless excitatory inputs from PPTN are present. This shunting mechanism is described by Equation (18) governing the membrane potential of the single unit in VP:

(18) $$\tau \cdot \frac{dm(t)}{dt}+m(t)=g_{\text{exc}}(t)-\Delta_{\Gamma }\left(g_{\text{exc}}(t)\right)\cdot g_{\text{inh}}(t)+B+\eta (t)$$

where τ = 10 ms, *B* = 0.5 is the baseline activity of the VP neuron and Γ = 0.1 is a threshold on excitatory inputs. The inhibitory projection from NAcc is learned according to the thresholded Hebbian learning rule described by the Equation (7).

#### 2.2.9. Lateral habenula

LHb is activated by aversive stimuli and reward omission (Hikosaka et al., 2008; Hong et al., 2011). In this model, signaling of reward omission is provoked by disinhibition from VP: when VP is inhibited by NAcc at the expected time of reward delivery, it stops inhibiting LHb and allows it to fire. As the source of excitatory inputs to LHb is still not clear, we simply consider in this model that the single LHb cell has a very high baseline activity, which is normally canceled by the tonic inhibition of VP, as expressed by Equation (19):

(19) $$\tau \cdot \frac{dm(t)}{dt}+m(t)=-g_{\text{inh}}(t)+B+\eta (t)$$

with τ = 10 ms and *B* = 1.

#### 2.2.10. Rostromedial tegmental nucleus

While most RMTg neurons are activated by aversive events, some also respond to reward omission. They are inhibited by rewards and reward-predicting stimuli (Jhou et al., 2009). The excitation at reward omission has been shown to come from LHb glutamatergic inputs (Balcita-Pedicino et al., 2011; Hong et al., 2011). In this model, the single unit of RMTg is under the tonic inhibition from VP (Jhou et al., 2009), and can become activated when excitatory inputs from LHb are present, as formulated by the Equation (20):

(20) $$\tau \cdot \frac{dm(t)}{dt}+m(t)=g_{\text{exc}}(t)-g_{\text{inh}}(t)+\eta (t)$$

with τ = 10 ms.

#### 2.2.11. Ventral tegmental area

The final stage of the model is a single dopaminergic unit in VTA. It receives excitatory inputs from PPTN, inhibitory inputs from RMTg and modulatory inhibitory inputs from NAcc. The excitatory inputs can progressively be canceled by the modulatory inputs, as the US becomes temporally predictable by NAcc. Additionally, RMTg inputs can provoke a prolonged inhibition of the VTA cell below baseline if no reward is present. This is reflected by the Equation (21):

(21) $$\begin{array}{l} \tau \cdot \frac{dm(t)}{dt}+m(t)=g_{\text{exc}}(t)*\left(1-\Phi_{\tau_{\text{mod}},k}\left(g_{\text{mod}}(t)\right)\right)-\\ \;\left(1-\Delta_{\Gamma }\left(g_{\text{exc}}(t)\right)\right)\cdot \Phi_{\tau_{\text{inh}},k}\left(g_{\text{inh}}(t)\right)+B+\eta (t) \end{array}$$

with τ = 10 ms, τ_mod_ = 300 ms, *k* = 1, Γ = 0.1, τ_inh_ = 30 ms, and *B* = 0.2. Modulatory inputs from NAcc are learned according to the learning rule defined in Equation (7).

## 3. Results

Most experiments in this section concern the concurrent learning of the three CS-US associations described in Table 1. The learning procedure is split into two phases: the sensitization phase, where each US is presented alone for 10 trials, and the conditioning phase, where the CS and US are presented together for 15 trials. The three CS-US associations are intermingled in ascending order for simplicity, but a randomized order would not change the results. The organization of each trial is described in section 2.2.3.

### 3.1. CS-US associations in the amygdala

**Figure 4:**
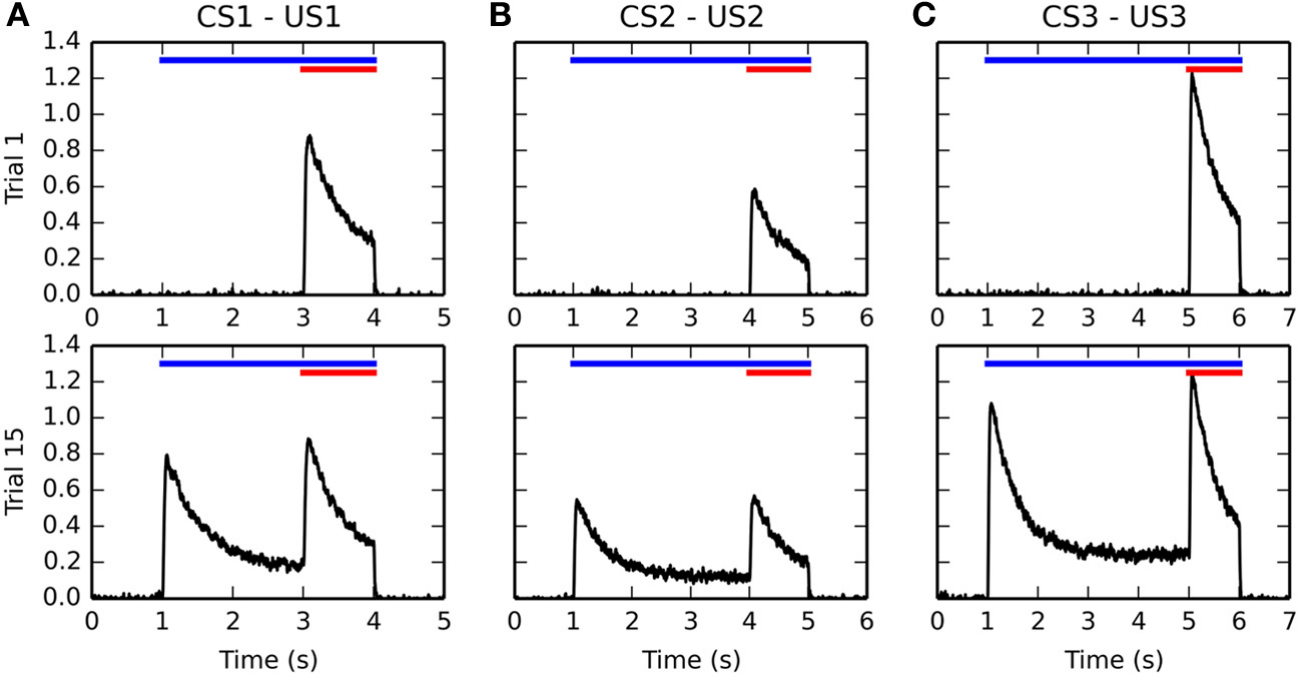
**Timecourse of the activity of different BLA cells before and after conditioning**. Activities for the CS1-US1, CS2-US2, and CS3-US3 associations are represented from left to right in panels **(A–C)**, respectively. For each figure, the horizontal blue line represents the presentation of the CS, while the red line represents the presentation of the US. The top row shows the evolution of the firing rate of a single BLA neuron over time during the first trial of conditioning. Because of the sensitization phase and the lateral inhibition in BLA, there is only one cell in the population which represents each US. During the first trial, this cell gets maximally activated at the time of reward delivery (3, 4, and 5 s after the start of the trial, respectively), and its firing rate decreases because of the adaptation of excitatory inputs in BLA, before returning to baseline when the US is removed after 1 s. All other cells in BLA are not activated. The bottom row shows the activity of the same cells during the 15th trial of conditioning. They now show an increase of activity when the CS appears (1 s after the start of the trial), reaching a maximum of similar amplitude as the response evoked by the US, and slowly decreasing to a baseline of about 20% of this maximal activity. When the reward is delivered, they increase their firing rate similarly a in the first trial.

Figure 4 shows the firing rate of single BLA cells during the 1st (top row) and 15th (bottom row) trials of the conditioning phase, for each of the three CS-US associations. After the sensitization phase, only one cell in BLA is selective for each US because of the increased competition induced by antihebbian learning in the lateral connections within BLA. The activity of these US-specific neurons only is displayed, the other cells having a firing rate close to 0.

During the first conditioning trial, each BLA cell is activated only at reward delivery, with an amplitude proportional to the magnitude of the US. It reaches a peak shortly after US onset and slowly decreases to a small baseline because of the phasic integration of LH inputs described in Equation (10). During the late conditioning trial, the same cells are activated by the onset of the corresponding CS. Their firing rate also reaches a peak shortly after CS onset, with a magnitude proportional to the reward magnitude (see section 3.4 for further discussion) and slowly decays to around 20% of their peak amplitude, due to the temporal integration of IT inputs in Equation (10). However, these cells are still phasically excited by the delivery of the predicted reward.

This behavior of single BLA cells during conditioning is in agreement with the known dependency of BLA activity on reward magnitude (Bermudez and Schultz, 2010) as well as with the observed firing rate of individual BLA neurons for both CS and US (Ono et al., 1995; Maren and Quirk, 2004). As CE simply sums up BLA activity in our model, the response profile in CE is similar during conditioning, although not specific to the CS-US association. This means that the CE → PPTN → VTA pathway is able to signal the onset of specific reward-predicting cues to VTA and generate the corresponding phasic burst, as observed experimentally (Lokwan et al., 1999; Fudge and Haber, 2000).

### 3.2. Timecourse of activity in VTA

**Figure 5:**
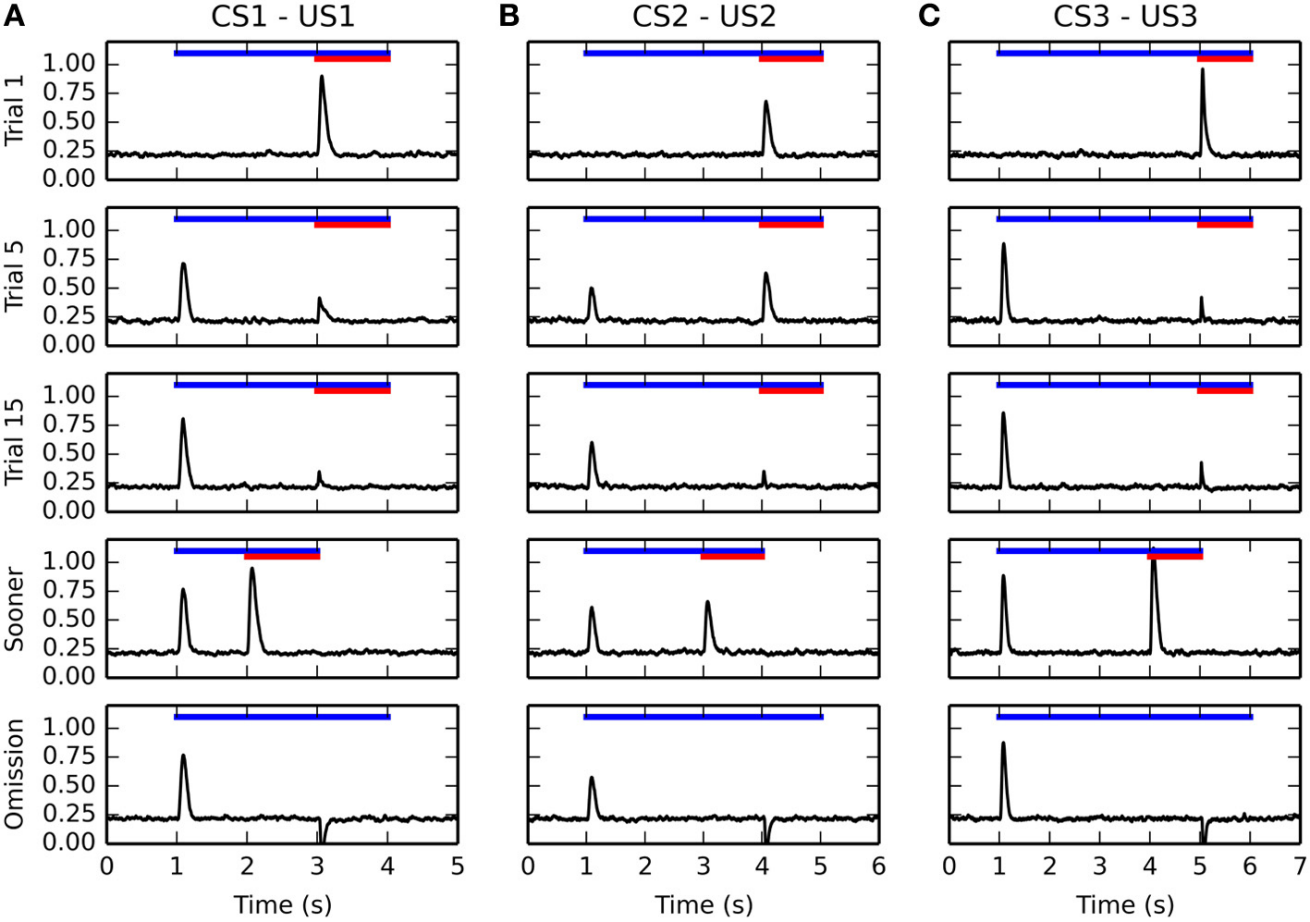
**Timecourse of the activity of the VTA cell during conditioning**. The activity for the three CS-US associations is displayed from left to right in panels **(A–C)**, respectively. For each figure, the horizontal blue line represents the presentation of the CS, while the red line represents the presentation of the US. The first row represents the activity of VTA during the first trial of conditioning, the second row during the 5th trial, the third during the 15th trial. They show a progressive reduction of the amplitude of the US-related burst, while the CS-related burst appears early in learning. The fourth row shows the activity of the VTA cell when the reward is delivered 1 s earlier than previously associated. It shows that the VTA cell responds to rewards delivered earlier with the same activation as for unpredicted rewards. The fifth row shows omission trials: the CS is presented normally, but the US is omitted. The VTA cell shows a phasic pause in firing at the time when reward was expected.

Figure 5 shows the temporal evolution of VTA activity during several conditioning trials for the three CS-US associations. The first row shows its activity during the first conditioning trial. As expected, the VTA cell only fires transiently at reward delivery, with an amplitude proportional to the reward magnitude. This phasic excitation is provoked by the LH → PPTN → VTA pathway.

The second and third rows show VTA activity during the 5th and 15th conditioning trials for each association. The DA cell shows very early in learning a phasic burst of activity at CS onset. In parallel, the amplitude of the US-related burst progressively decreases until an almost complete cancelation at the 15th trial. This pattern of evolution is in accordance of the observations of Pan et al. (2005) showing that the CS- and US-related bursts of DA activation coexist in the early phases of training. Simple disconnection experiments show that the CS-related phasic bursts are dependent on the CE → PPTN → VTA pathway, while the cancelation of the US-related bursts is dependent on the modulatory projection from NAcc to VTA.

After 15 conditioning trials for each association have been executed, two additional trials are performed to test the functional properties of the model. The first additional trial (fourth row of Figure 5) consists in early delivery of reward: the US previously paired with the CS is presented 1 s earlier than usual (i.e., 1 s after CS onset instead of 2 s for the CS1-US1 association, 2 s for CS2-US2, and 3 s for CS3-US3). The CS presentation stops with the end of the US. In this case the VTA cell reacts phasically to reward delivery with the same amplitude as for an unpredicted reward, instead of the diminished burst observed when the reward is presented at the expected time. This is in accordance with the experimental findings of Hollerman and Schultz (1998).

In the second type of additional trial (fifth row of Figure 5), each CS is presented normally but the US is omitted. Shortly after the expected delivery time (around 50 ms), the VTA cell receives a strong phasic inhibition bringing its firing rate to 0 for a prolonged period of time. This activation dip is provoked by the NAcc → VP → LHb → RMTg → VTA pathway. This behavior is in accordance with the reward-prediction error interpretation of VTA activity during conditioning (Schultz et al., 1997; Fiorillo et al., 2003).

### 3.3. Evolution of VTA activity during conditioning

**Figure 6:**
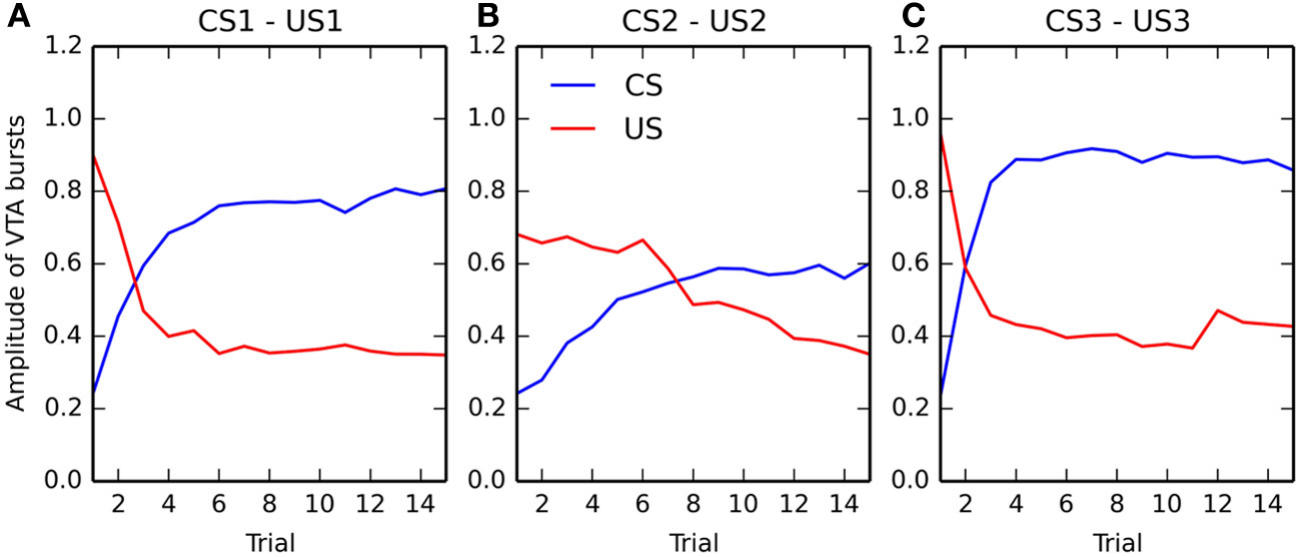
**Evolution of the maximal activity in VTA during conditioning**. For each of the three associations (panels **(A–C)**, respectively), the maximal activity of the VTA cell at CS onset (in blue) and at reward delivery (in red) is plotted for each trial of the conditioning phase. These values are computed by taking the maximum value of the firing rate of the VTA cell in a small time window (±100 ms) around CS onset and reward delivery. The panels show the relative speed at which the CS-related bursts appear and the one at which the US-related bursts are canceled.

In this section, we take a closer look at the evolution of phasic activities in VTA during the conditioning process. Figure 6 shows the evolution of US- and CS-related activation in BLA over the 15 conditioning trials, for each of the three associations. The amplitude of the CS-related (in blue) and US-related (in red) bursts is computed by taking the maximal firing rate of the VTA cell in a small time window (±100 ms) around CS and US onsets, respectively.

Panels **(A)** and **(C)** (corresponding to rewards of magnitude 0.8 and 1.0, respectively) show that the CS-related bursts, initially non-existent as the baseline activity of VTA is 0.2, quickly rise in a few trials to reach up a limit dependent on the reward magnitude. The US-related bursts show the opposite pattern: the amplitude is initially dependent on the reward magnitude, but is progressively decreases to a value close to the VTA baseline. One can observe that the cancelation is not total, the maximal value of US-related bursts being between 0.3 and 0.4, while the baseline activity is 0.2. However, the duration of the phasic is also reduced from approximately 200 ms for unpredicted rewards to 50 ms for fully predicted rewards, so the total amount of dopamine released can be considered relatively low. This aspect will be discussed in section 4.2.

Panel **(B)**, corresponding to a reward magnitude of 0.5, shows a different behavior. While the CS-related burst still increases to reach a maximum equal to the initial US-related burst (although more slowly), the cancelation of the US is both slower and not total. This suggests that reward magnitude influences conditioned responses in VTA in a non-linear manner. This will be further investigated in the following section. Altogether, the results show that the cancelation of the US-related VTA activation happens well after the appearance of CS-related bursts, what is consistent with the experimental data (Pan et al., 2005).

### 3.4. Influence of reward magnitude on conditioning

**Figure 7:**
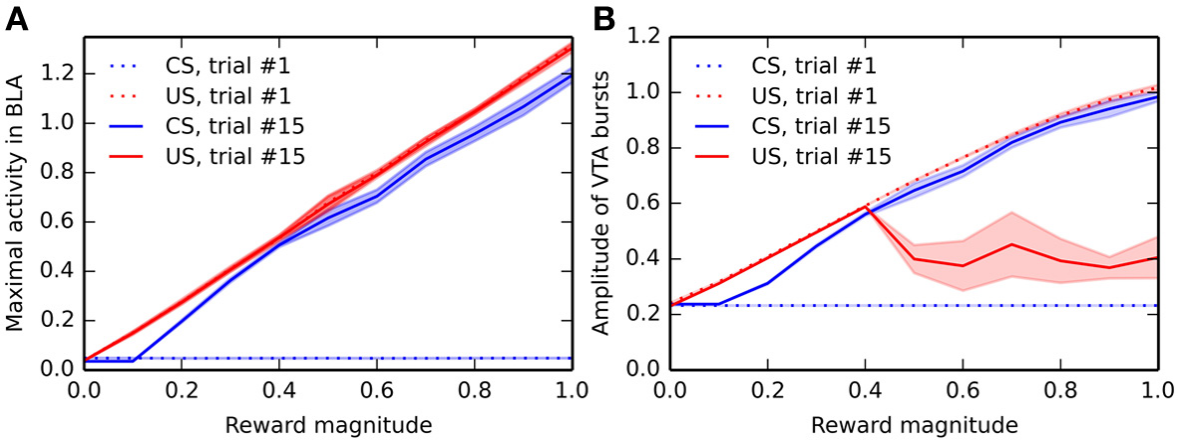
**Dependency of the activity in BLA and VTA on reward magnitude**. Panel **(A)** shows the maximal firing rate in BLA around CS-onset and reward delivery during the first and last trial of conditioning, for different reward magnitudes. For each value of the reward magnitude, the CS1-US1 association is presented 15 times, and the maximal activity in BLA around CS-onset (between 900 and 1100 ms after the start of each trial) and reward delivery (between 3900 and 4100 ms after the start of the trial) is recorded. The experiment is repeated 10 times (without different initial values), and the mean (solid line) and standard deviation (colored area) of these measurements are plotted. The blue dotted line shows the maximal activity at CS-onset during the first trial, which does not depend on reward magnitude, as no learning has taken place yet. The red dotted line shows the maximal activity at reward delivery during the first trial, which is proportional to the reward magnitude because of learning in the LH → BLA projection during the sensitization phase. For the last trial of conditioning, the blue and red solid lines show the dependency on reward magnitude of the maximal activity in BLA at CS onset and reward delivery, respectively. While the US-related response is proportional to the reward, the CS-related activity only appears for reward magnitudes bigger than 0.1. Panel **(B)** shows the dependency on reward magnitude of the VTA bursts in the same conditions (blue dotted = CS onset at trial 1, red dotted = US delivery at trial 1, blue solid = CS onset at trial 15, red solid = US delivery at trial 15). While there are no CS-related bursts during trial 1, the US-related burst is proportional to reward magnitude. A similar relationship can be observed for the CS-related burst at the end of learning. However, the US-related burst after learning shows a different pattern: small rewards (magnitude smaller than 0.4) elicit burst proportionally to their magnitude, but bigger rewards elicit strongly attenuated bursts, showing that the cancelation of US-related bursts is dependent on reward magnitude.

In order to study the influence of reward magnitude on VTA activity, we modified the conditioning procedure. In this section, only one CS-US association (CS1-US1, with an interval of 2 s between the CS and US) is learned by the network, but the reward magnitude is varied linearly between 0 and 1 instead of the previous value 0.8. For each value of the reward magnitude, a different network performs the sensitization and conditioning tasks for this particular association. Activities in BLA and VTA are recorded during the 1st and 15th conditioning trials, and the maximal activity of VTA and BLA cells at CS and US onsets (computed within a time window of ±100 ms) is shown on Figure 7, averaged for 10 different networks. Figure 7A shows the dependency of US- and CS-related activation in BLA on reward magnitude, while Figure 7B shows the reward-magnitude dependency of VTA bursts.

During the first trial of conditioning, there is logically no CS-related activity in BLA and VTA (blue dotted line), regardless the reward magnitude, as conditioning has not taken place yet. The US-related activity (red dotted line) shows a linear dependency on reward magnitude in both VTA and BLA. This is explained by the linear encoding of reward magnitude in LH: a more precise model of food-related activation in LH may change this property.

During the last trial of conditioning, the CS elicits strong phasic activity in both BLA and VTA (blue solid line), which is roughly proportional to the reward magnitude: additive noise plays an important role in the learning dynamics of the model, what explains that different networks may exhibit slightly different results. This is in accordance with the observation that CS-elicited DA bursts increase monotonically with the magnitude of expected rewards (Tobler et al., 2005).

The situation is more contrasted regarding the US-related activation after conditioning (red solid line): while BLA still phasically responds linearly to the US magnitude (see also Figure 4), the cancelation of reward-delivery bursts in VTA only occurs if the reward magnitude is high enough (above 0.4). This cancelation is dependent on learning in NAcc, which is itself dependent on DA release by VTA. Small rewards do not provoke sufficiently high VTA bursts to modulate striatal processing and learning. While there is no direct evidence of such an effect of reward magnitude on US cancelation, this effect is in agreement with the known influence of reinforcer magnitude on the emergence of conditioned responding (Morris and Bouton, 2006) or peak-interval tasks (Ludvig et al., 2007), which are dependent on learning in the striatum.

### 3.5. Timing mechanism in NAcc

An important functional aspect of the model is the inducement of dips in VTA when a predicted reward is omitted. It relies on the ability of specific NAcc cells to learn the CS-US interval duration based on inputs from the synchronized oscillators in vmPFC, gated by the dopaminergic bursts of VTA.

**Figure 8:**
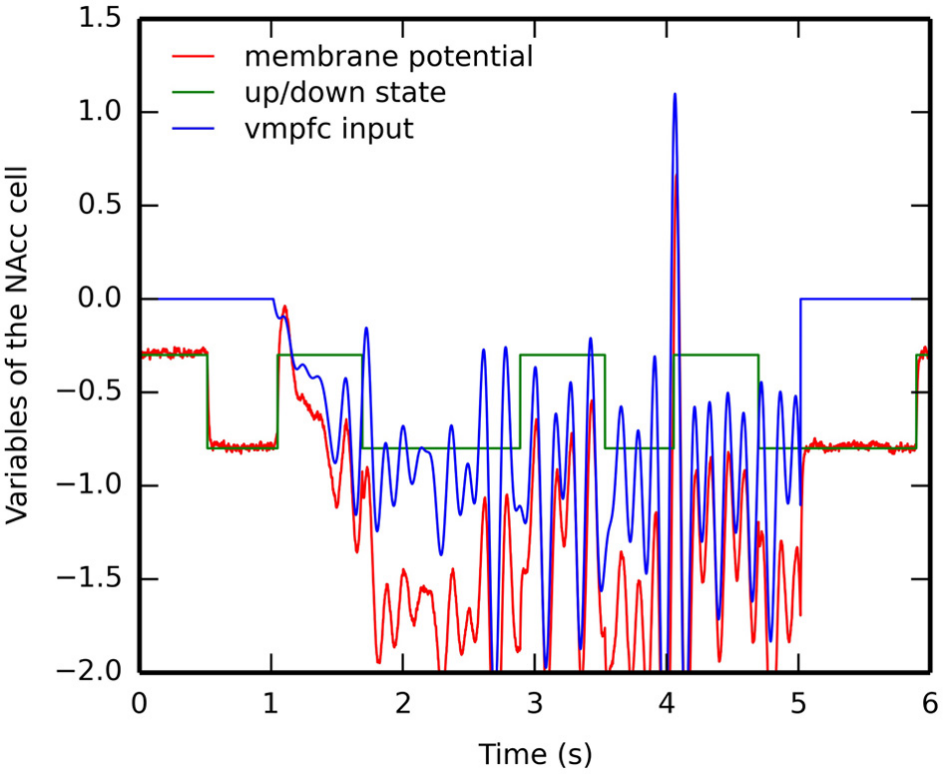
**Timecourse of the internal variables of a single NAcc neuron during a reward omission trial**. After the conditioning phase, CS2 is presented alone. The NAcc neuron which was selective for US2 during conditioning is recorded: its membrane potential *m*(*t*) in red, the weighted sum of excitatory inputs from vmPFC in blue and its up- or down-state *s*(*t*) in green. The firing rate of the neuron is the positive part of the membrane potential: the firing rate becomes only non-zero shortly at the time where reward is expected but omitted.

Figure 8 shows the evolution of several internal variables of one NAcc cell during reward omission. This cell is selective for the US2 because of the corresponding input from BLA. After successful learning of the CS2-US2 association (15 trials), CS2 is presented alone while we record the temporal evolution of (1) the membrane potential of this cell (governed by Equation 16, red line), (2) the weighted sum of excitatory inputs from vmPFC (blue line) and (3) its up- or down-state *s*(*t*) (green line). For simplicity, its firing rate is not depicted, as it is only the positive part of the membrane potential.

When the CS appears 1 s after the start of the trial, the CS-evoked VTA burst brings the cell into the up-state, while the cortical oscillators start influencing the membrane potential. However, this excitation is not sufficient to bring the membrane potential above the threshold and activate the cell. During the delay period, the cell switches between down- and up-states based on the internal dynamics of the variable *s*_time_(*t*) (Equation 17). The sum of inputs from vmPFC oscillate during this period, but is never strong enough to activate the cell. However, at the time when the US is expected (4 s after the beginning of the trial), these inputs become able to bring the cell into the up-state, what results in a membrane potential well above threshold and provokes a short burst of the firing rate, although the US is not delivered.

This mechanism is very similar to the Striatal-Beat Frequency model proposed by Matell and Meck (2004), although based on a different implementation (different number of cortical oscillators, different frequency range and different learning rule). The weighted sum of cortical inputs, which peaks for the cortical pattern describing the learned interval, fluctuates a lot during the delay period. In particular, there are several peaks during the delay period corresponding to different harmonics ($\frac{1}{2}$, $\frac{1}{3}$,…,). As suggested in Matell and Meck (2004), the up- and down-states are necessary to avoid spurious activation of NAcc during this period, what would lead to unwanted VTA dips, especially at the beginning of learning. In the early conditioning trials, the vmPFC input is too weak to bring the NAcc cell into the up-state, which is only dependent on phasic DA bursts at reward delivery. As in the Striatal-Beat Frequency, we do not precisely model how the cortical oscillators could be synchronized at CS onset: it is a simple threshold on visual inputs from IT. A more detailed model is necessary to generate these oscillations, perhaps through the opening of a vmPFC → ventral BG → medial thalamus → vmPFC loop, gated by the VTA burst at CS onset.

### 3.6. Acquisition rate of temporal prediction

In order to study the speed at which the CS-US interval is learned in NAcc, we designed a different conditioning schedule. After sensitization to the three US, the 15 conditioning trials per association are alternated with omission trials, i.e., each CS-US trial is immediately followed by the CS alone. All learning rules are disabled during these omission trials, as we only want to use the CS as a probe to measure the acquisition rate: we want to study what would happen if the reward were omitted earlier in the conditioning process.

**Figure 9:**
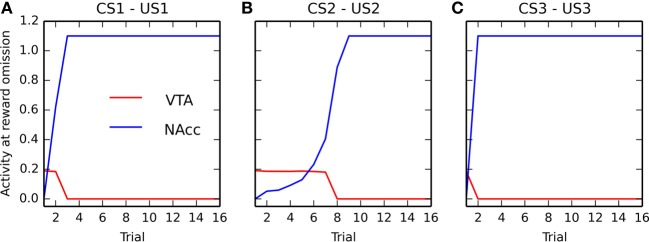
**Apparition of VTA dips during conditioning**. For the three CS-US associations (panels **(A–C)**, respectively), the panel represents what would happen in VTA (red) and NAcc (blue) if the reward were omitted directly after each conditioning trial. Learning is shut off during these omission trials. The red line shows the minimal activity in VTA during these omission trials. After the first few conditioning trials, this minimal activity is around the baseline (0.2), but quickly becomes equal to 0, denoting the appearance of the strong phasic inhibition of VTA at reward omission. The blue line shows the emergence of activity in NAcc at reward omission. The speed at which the timing prediction appears in the ventral BG depends on reward magnitude.

Figure 9 shows the maximal activity in NAcc (blue line) and the minimal activity in VTA (red line) during these omission trials for each CS-US association **(A–C)**. One can observe that NAcc becomes quickly able to react for an omitted reward (after only two conditioning trials for CS3, three for CS1 and seven for CS2). The speed of learning is therefore dependent on reward magnitude, what is due to the dopaminergic modulation of cortico-striatal learning: smaller rewards generate smaller VTA bursts, inducing less LTP in the NAcc. The VTA dips are directly dependent on this learning: as soon as NAcc is able to get activated for omitted rewards, the minimal activity in VTA at reward omission switches from the VTA baseline activity (0.2) to 0, indicating that VTA successfully signals reward omission.

This result is in accordance with experiments showing that the time interval from CS onset to US delivery is learned very rapidly at the start of training (Balsam et al., 2002). Although reward magnitude was long considered as playing only a minor role in acquisition speed during conditioning (Gallistel and Gibbon, 2000), more recent experiments showed that it influences the number of trials needed by an animal to exhibit conditioned responses during both appetitive and aversive conditioning (Morris and Bouton, 2006) and that it speeds up learning of discrimination tasks (Rose et al., 2009). In accordance with these results, our model predicts that the ability to signal negative reward-prediction errors is learned faster when the reward magnitude is high.

### 3.7. Time course of forebrain nuclei

**Figure 10:**
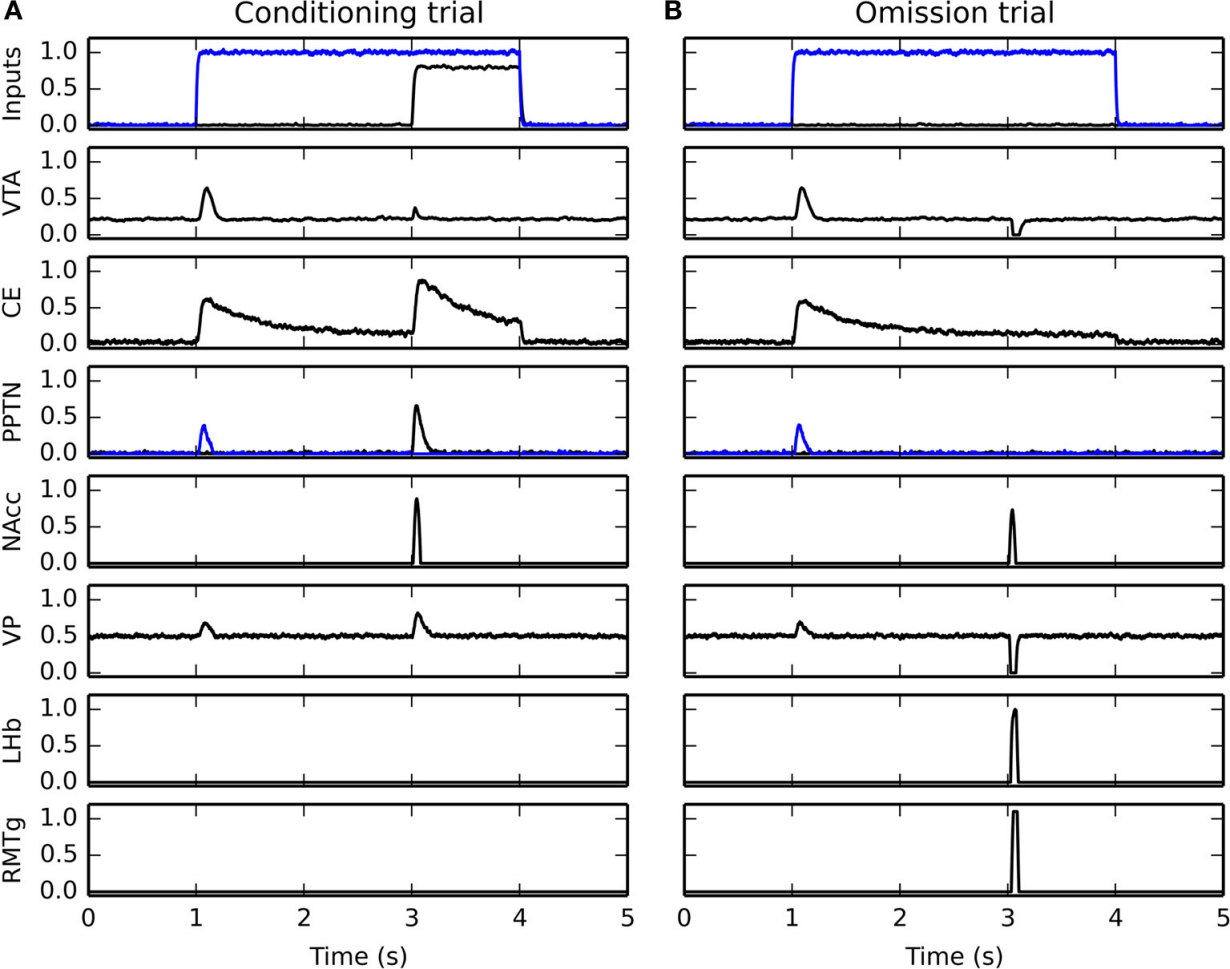
**Timecourse of activity in different areas of the model**. Panel **(A)** shows the activity during the last conditioning trial of the CS1-US1 association, while panel **(B)** shows what happen during reward omission after learning (CS1 alone). The first row shows the inputs to the network, with the blue line showing the mean activity in the IT cluster corresponding to CS1, while the black line shows the mean activity for the neurons of LH representing US1. The second row shows the timecourse of the VTA cell during these trials, similar to what is shown on Figure 5. The third row shows activity in CE, which matches the already observed timecourse in BLA during conditioning on Figure 4. The fourth row depicts the timecourse of activity in PPTN, with the blue line showing the unit responding to CS onset (with inputs from CE) and the black the one responsive the US (with inputs from LH). The fourth, fifth, sixth, seventh, and eighth rows depicts the maximal activity in NAcc, VP, LHb, and RMTg, respectively. Please refer to the text for how these activations relate to each other.

In order to better understand how the different nuclei in the model interact during conditioning and reward omission, Figure 10 shows the time course of activity of several populations during the 15th conditioning trial of CS1-US1 (Figure 10A), followed by the omission of US1 (Figure 10B). The first row depicts the inputs to the networks, with the blue line showing the mean activity in the IT cluster selective for CS1 and the black line showing the mean activity of the LH neurons representing US1. As previously shown, VTA (second row) exhibits a phasic burst at CS onset on both trials, but barely reacts after learning when the reward is delivered, while it is strongly inhibited when the reward is omitted. The CS-driven burst is due to associative learning in the amygdala, what is reflected in the activity of the CE unit (third row). The transient activation of CE excites the CS-selective population in PPTN (fourth row, in blue), which in turn generates the phasic VTA burst and excites VP (sixth row). The excitation of VP increases the inhibition on LHb (seventh row) and RMTg (eighth row), which therefore remain silent.

When the reward is delivered (Figure 10A), LH activates directly the US-selective population of PPTN (fourth row, in black), but also the amygdala (reflected in the excitation of CE). However, the strong competition between the CS- and US-related populations of PPTN results in the phasic activation of the US group only (as it receives LH inputs slightly before the CS group gets activated by CE, which is a disynaptic pathway and therefore slower). The US group of PPTN activates VTA and VP similarly. At the same time, NAcc gets activated by the reward delivery, through its inputs from BLA and vmPFC, in conjunction with the phasic VTA burst bringing the cell into the up-state. NAcc is then able to cancel the VTA burst through its direct modulatory projection. NAcc also inhibits strongly VP, but this inhibition is canceled by the excitatory projection from PPTN to VP. VP therefore keeps inhibiting LHb and RMTg, and no VTA dip is observed.

When the reward is omitted (Figure 10B), PPTN does not receive inputs from LH or CE. The activation of NAcc at the expected time of reward delivery is now able to inhibit strongly VP, what releases LHb and RMTg from its strong tonic inhibition. LHb becomes transiently activated, exciting RMTg which can provoke a complete pause in VTA firing.

Although not directly comparable to recorded firing rates, the displayed time courses are in agreement with several observations, such as the activation of two different populations of PPTN neurons for reward-predictive cues and rewards (Pan and Hyland, 2005), the activation at reward omission of LHb (Hikosaka et al., 2008; Hong et al., 2011) and RMTg (Jhou et al., 2009), or the activation of VP for large reward-predicting cues and rewards (Tindell et al., 2004; Smith et al., 2009). VP is also inhibited at reward omission, what is consistent with the observed inhibition of some VP cells when small rewards is received during a session where larger rewards are available (Tachibana and Hikosaka, 2012).

## 4. Discussion

We have proposed a neuro-computational model of the afferent system to the dopaminergic area VTA, which is able to reproduce several observations on VTA's behavior during appetitive conditioning: progressive appearance of phasic bursts of activity at CS onset, progressive diminution of the amplitude of the phasic bursts elicited by primary rewards, strong phasic inhibition at the time when reward is expected but not delivered (Schultz et al., 1997; Fiorillo et al., 2003; Pan et al., 2005). Cancelation of US-related bursts and inhibition at reward omission both rely on learning of the duration of the CS-US interval in the NAcc, which influences VTA either directly or through the output structures of the ventral BG. This is in accordance with experiments showing that rewards delivered earlier than expected provoke a very high amplitude VTA burst which would have been canceled if delivered at the learned time (Hollerman and Schultz, 1998). Furthermore, the model reproduces the dependency on reward magnitude of the activities in BLA (Bermudez and Schultz, 2010) and VTA (Tobler et al., 2005).

There are several aspects of reward processing and dopaminergic activity which are not covered by this model: the model is limited in its current form to classical conditioning and does not specifically address instrumental conditioning or goal-directed learning. However, Pavlovian-to-Instrumental transfer of learning, which is known to be particularly dependent on NAcc, is thought to be a critical component of goal-directed learning (Cardinal et al., 2002; Corbit and Balleine, 2011) and the proposed model is a first step toward understanding these processes. Consequently, the model does not incorporate yet the known effects of the tonic component of VTA activity, which is thought to modulate motivation and engage reward-directed behaviors (Daw et al., 2006; Niv et al., 2007), and focuses only on the phasic components of VTA activity.

Three dimensions are particularly relevant in reward processing: reward magnitude, reward probability and time, with NAcc having been shown crucial in the adequate response to each of these dimensions (Stopper and Floresco, 2011). The proposed model focuses on reward-magnitude and time, leaving reward probability to further work. Manipulating reward probability will require to investigate the effect of VTA dips on learning in BLA and NAcc, with the extreme end of the spectrum being extinction of conditioning (Tye et al., 2010).

Within these validity boundaries, the model is able to make several testable predictions, among which the fact that VTA dips should only appear for sufficiently big rewards, or that the number of trials needed to observe US-related burst cancelation should be proportional to reward magnitude. It also predicts that at least a subpopulation of NAcc (presumably in the shell part) should be activated by reward omission. This prediction will be further discussed in the rest of the section.

From the neuro-computational point of view, the model is fully autonomous: it only learns from the relative timecourse of CS and US inputs. Apart from the distinction between the sensitization and conditioning phases, no additional mechanism such as a central executive is required to control learning in any of its populations. It relies only on the numerical integration of a set of interdependent dynamical equations, in conjunction with sensory inputs. Moreover, the neural mechanisms employed provide scalability, as multiple CS-US associations can be learned in parallel, depending on the number of neurons in BLA and NAcc. Future work will address its integration on a neurorobotical platform with realistic inputs.

### 4.1. Relation to other work

Early implementations of the TD algorithm used a unitary backward chaining mechanism using serial-compound temporal representations of the CS, where the value of the reward is progressively transferred to the previous time step (or state), until it corresponds to CS onset (Montague et al., 1996; Schultz et al., 1997; Suri and Schultz, 1999). For each time step of the conditioning sequence, DA represents a reward prediction error, i.e., the discrepancy between the amount of predicted reward and the actually received reward. Unless very long eligibility traces are used, TD predicts that DA bursts will gradually shift backwards in time from reward delivery to CS onset, what is not observed experimentally (Pan et al., 2005). This also implies that the mechanism should work for any higher-order conditioning task, transferring the phasic burst to the earliest predictor of reward. In practice, only second-order conditioning has been observed, as noted in Hazy et al. (2010). It, however, explains phenomenologically many aspects of DA activity during conditioning and has been used with great success in action-selection and decision-making frameworks as long as the action space is not too large, but its mapping on brain structures is problematic.

Ludvig et al. (2008) introduced an alternative temporal representation of the stimuli for the TD(λ) algorithm. A set of overlapping temporal basis functions is used to filter out an exponentially decreasing trace of the stimuli (both CS and US) and provide a coarse coding of the time elapsed since stimulus onset. The output of this microstimuli representation gradually becomes weaker and coarser as time goes. Using these representations as inputs, the TD(λ) algorithm is able to learn a reward-prediction error signal, gradually responding positively to the CS while canceling its response to the US. If the US is omitted, it exhibits a negative reward-prediction error, although much weaker than previous versions of TD. If the reward is delivered earlier than expected, it responds maximally to it but shows only a very small dip at the expected time, without the need for an explicit reset of the temporal representations (see below for a discussion). A later extension of this model (Ludvig et al., 2009) incorporated an additional array of microstimuli signaling the presence of a stimulus in addition to its trace and was able to better explain the functional difference between delay and trace conditioning, as well as to make interesting predictions about the role of the hippocampus in trace conditioning.

The model of Rivest et al. (2010, 2013) used an interesting approach to provide a temporal representation of the stimuli to the TD(λ) algorithm: a LSTM network (Hochreiter and Schmidhuber, 1997) is used to learn a temporal representation of both CS and US based only on stimulus onset and the reward-prediction error signal. A LSTM network is composed of recurrent memory blocks, each integrating its inputs depending on an adaptive gating function. This allows to learn to represent the CS by ramping functions peaking just before US delivery, allowing the TD(λ) to access an adaptively timed representation of the stimulus. This model exhibits all the expected temporal properties of the DA signal in both delay and trace conditioning without any explicit representation of the task. Although needing an irrealistic number of trials to converge and having a significant error rate, this model builds an interesting bridge between reward-prediction, timing and working memory processes.

The proposed model shares more assumptions with the dual-pathway models. The model of Brown et al. (1999), later extended by Tan and Bullock (2008), has been a very important step in overcoming the problems of TD, and many of its assumptions still hold true. It similarly considers that rewards provoke DA bursts (although in SNc rather than VTA, but this is more a labeling issue) through the LH → PPTN → SNc pathway. Reward-predicting cues progressively elicit burst firing through the NAcc → VP → PPTN → SNc pathway, while the striosomes of NAcc learn to generate lagged, adaptively timed signals inhibiting SNc at the time when reward is expected. The comparison between the predicted and received rewards occurs directly at the level of the dopaminergic cells, while it occurs in VP in our model, providing an explanation for the role of LHb and RMTg in reward omission. Moreover, this model hypothesizes a common NAcc → SNc pathway for both US-related burst cancelation and dips at reward omission, while they are functionally separated in our model. The major problem with the model of Brown et al. (1999) and Tan and Bullock (2008) in our view is the mechanism underlying the adaptively timed inhibitory learning in the striosomes of NAcc. The proposed intracellular spectral timing mechanism (Grossberg and Schmajuk, 1989; Fiala et al., 1996), relying on mGLUR1-mediated delayed Ca^2+^ spikes with distinct time constants for each striosomal cell, indeed allows to learn specific duration in conjunction with DA bursts, but the maximal interval learnable by this mechanism is equal to the longest delayed spike possible, what is likely to lie in the sub-second range as in the cerebellum (Fiala et al., 1996). For the supra-second range, network-based oscillatory mechanisms such as the striatal-beat frequency model are more likely to be sufficiently efficient and robust to learn such delays (Coull et al., 2011).

The model called PVLV (Primary-Value and Learned-Value) initially proposed by O'Reilly et al. (2007) and refined in Hazy et al. (2010) builds up on these ideas. The primary value (PV, the value of the reward itself) and the learned value (LV, the value of the reward-predicting cue) during conditioning are computed by two different afferent systems to VTA, both with an excitatory and an inhibitory component. The excitatory PV system PVe signals reward delivery to VTA through a direct connection from LH to VTA, although a relay through PPTN would perform the same function as in our model. The excitatory LV system LVe learns to generate DA bursts at CS onset, through a direct projection from CE to VTA: as in our model, the amygdala learns to associate a sustained representation of the CS to the delivery of reward when the US-related burst (or dip) occurs. The inhibitory PV system PVi, composed of the striosomal neurons in NAcc, learns to cancel progressively US-related bursts, but in an almost time-independent manner: they use a ramping function activated by CS onset and peaking at reward delivery that modulates the reward prediction. The origin of such as signal is putatively in the cerebellum, but no details are provided on how such a signal could be adapted to different CS-US durations. Moreover, this implies that rewards given earlier than expected would still provoke attenuated DA bursts. Last, the inhibitory LV system LVi, also in the striosomes of NAcc, slowly learns to cancel CS-related bursts in order to avoid over-learning in auto-shaping experiments (where the CS becomes an incentive to action, what is not covered by our model). The main issue with this model is that timing mechanisms are only phenomenologically incorporated, what may be due to the fact that the equations governing neuronal activation and learning are discretized with a time step of 1 s, instead of 1 ms in the model of Brown et al. (1999) or ours. However, this model explains several aspects of conditioning, including acquisition, extinction, blocking, overshadowing, conditioned inhibition and second-order conditioning. Furthermore, it has been successfully integrated into a wider functional model of working memory including the prefrontal cortex and the dorsal BG (O'Reilly and Frank, 2006).

Together with an extensive review of the functional and electrophysiological properties of the ventral basal ganglia, Humphries and Prescott (2010) propose a neuro-computational model of how a specific subcircuit of the ventral BG, involving the shell part of NAcc (which integrates cortical, amygdalar, and hippocampal inputs) and some part of VP, can selectively produce either bursts or dips in VTA, depending on the relative balance between the direct pathway (arising from NAcc cells carrying D1 receptors and projecting directly on VTA) and the indirect pathway (with NAcc neurons carrying both D1 and D2 receptors and projecting mainly on VP). In this framework, the prediction of a reward activates the direct pathway, what can either reduce the bursting amplitude or produce a dip in VTA, while the actual receipt of that reward activates the indirect pathway, canceling the influence of the direct pathway and allowing VTA bursts. While being more precise than our model on the functional role of NAcc cell subtypes, this model is limited to bursts or dips occurring at reward delivery (or at the time when reward is expected), but does not address the case of reward-predicting stimuli nor the issue of timing. This model has nevertheless the advantage of being understood equally well in the reward-prediction error framework of DA activity and in the action-outcome repertoire framework, which proposes that DA bursts primarily help associating an action with its delayed consequences (Redgrave et al., 2008).

Chorley and Seth (2011) proposed a dual-pathway model incorporating some concepts of the striatal-beat frequency model. It is composed of several populations of spiking point-neurons, subject to synaptic plasticity using a dopamine-modulated spike-timing dependent plasticity (STDP) learning rule (Izhikevich, 2007). In this model, the sensory representation of the US initially activates the DA population through an excitatory relay [either the subthalamic nucleus (STN) or the superior colliculus]. The corresponding DA burst enables STDP learning between the sustained sensory representation of the CS and STN, what leads to a progressive bursting behavior in VTA at CS onset. In parallel, the inhibitory pathway to VTA, involving the prefrontal cortex and the striatum, learns to progressively cancel the US-related burst and, if reward is omitted, to strongly inhibit the VTA population. The mechanism for learning the CS-US interval is similar to the striatal-beat frequency hypothesis: CS onset activates a pre-recorded sequence of spikes in the prefrontal cortex (identical in each trial) and the striatum learns to react phasically to the precise pattern corresponding to the elapsed duration at US onset. This pre-recorded sequence of spikes is functionally equivalent to a set of neural oscillators synchronized at CS onset and expressing reproducible patterns at the population level. Oprisan and Buhusi (2011) investigated a similar mechanism using Morris–Lecar neurons and showed that even noisy oscillators, with variable inter-spike intervals, are able to produce a population code for the elapsed duration since CS onset which can be detected by striatal coincidence detectors. The model of Chorley and Seth (2011) is an elegant mechanism describing the evolution of DA bursts during conditioning as well as for earlier delivery of reward or reward omission. It does not, however, map very precisely on the brain's architecture, nor take the effect of reward magnitude into account.

### 4.2. Biological plausibility

The structure of the proposed model is derived from known anatomical connections, and the used neural mechanisms are consistent with experimental data, either at the cellular or population level. It provides a minimal description of the network involved in controlling VTA activity during classical conditioning, with respect to a limited set of observations. However, there exists a certain number of other brain areas which are directly or indirectly involved in this process. Similarly, alternative mechanisms, especially for timing, might replace or complement the proposed ones. The purpose of this section is to discuss alternatives to the current assumptions.

One key assumption in the model is that there exists a subgroup of NAcc neurons, presumably in the striosomes (group of striatal neurons that project directly on SNc or VTA), which get activated at reward omission. The previously reviewed dual-pathway models also share this assumption, and justify it by observations that some cells in the ventral striatum display a ramping activity pattern, with firing rates almost linearly increasing from CS onset and peaking at the time when reward is expected (Schultz et al., 1992; Deadwyler et al., 2004). This indicates that the CS-US interval duration is indeed learned by NAcc cells, but raises the question of how such a ramping signal can be transformed into a phasic inhibition after reward is expected: direct inhibition of VTA by such ramping cells in NAcc should progressively reduce VTA firing as the time since CS onset increases, which is obviously not the case. Is there a still undiscovered group of NAcc cells firing only at reward delivery/omission, or do these ramping activities play a more complex role in the timing of CS-US intervals during conditioning? In the striatum, some cholinergic TAN interneurons show complex patterns (either excitation or inhibition) at reward omission (Apicella et al., 2009). As these cholinergic interneurons can disinhibit MSNs through the modulation of fast-spiking inhibitory interneurons and bring them in the up-state (Coull et al., 2011), it may provide a mechanism for the phasic activation of a subgroup of NAcc cells at reward omission. A more detailed model of the internal circuitry of NAcc is obviously needed.

Alternatively, ramping activities in the NAcc during the CS-US interval might complement or even replace such mechanisms. Such ramping activities have been also observed in the thalamus (Komura et al., 2001) and prefrontal cortex (Reutimann et al., 2004), with the slope of the ramp being proportional to the duration. This suggests that a cortex—ventral basal ganglia—thalamus loop might be a good candidate to actually learn the CS-US interval duration with climbing activities, modulated by the dopamine level. Based on this idea, many models have been proposed for interval timing using neural integration or drift-diffusion models (Durstewitz, 2004; Simen et al., 2011; Luzardo et al., 2013). The model of Rivest et al. (2010, 2013) is a good example of such a mechanism. However, how the maximal activity reached by such ramps is transformed into a precisely-timed phasic signal at reward omission still raises difficult technical questions, such as the effect of noise on the precision of neural integration, especially for long intervals, or the plausibility of the learning mechanisms.

In comparison to the other dual-pathway models, our model is to our knowledge the first to explicitly incorporate distinct origins for the cancelation of US-related bursts and for the dips at reward omission, although the idea was already proposed in Hazy et al. (2010) as a functional interpretation of the inhibitory component of the PV system PVi. As the authors noted, cancelation of a US-related burst must derive from an inhibitory signal occurring slightly in advance from the receipt of reward in order to be efficient, while the dips associated with omitted rewards occur clearly after the expected time, and the duration of these dips extends significantly longer than the corresponding bursts. They state that the first component is likely to be implemented by the direct inhibitory projection of NAcc on VTA, while the second results from a disinhibition of LHb by NAcc through a relay on VP, but the learning site of the CS-US duration is NAcc in both cases. This interpretation is consistent with our model. The question that arises is whether distinct subpopulations of NAcc participate in these two mechanisms: do the striosomes directly projecting to VTA exhibit ramping activity, thus being able to cancel US-related bursts in advance, while the matrix neurons, projecting to VP and therefore to the LHb/RMTg complex, exhibit a more phasic behavior and get activated only at reward delivery or omission, as predicted by the striatal-beat frequency model?

As observed experimentally (Fiorillo et al., 2008), the cancelation of the US-related bursts becomes weaker when the CS-US interval increases. We are not aware of any study reporting a similar effect of the interval duration on dips at reward omission. If not, this may support the idea that two different mechanisms govern the two types of inhibition: neural integration becomes less precise when the duration increases, as it becomes more difficult to detect when the maximum of the slope is attained, while coincidence detectors are more robust, provided that the oscillators are not too noisy (Matell and Meck, 2004; Oprisan and Buhusi, 2011).

An open issue with the coincidence detectors hypothesis is that corticostriatal learning is potentiated by DA bursts at reward delivery. Typical bursts in VTA are relatively long (150–200 ms), what implies that cortical oscillators with a frequency superior to 5 or 6 Hz can show a full period during the burst. In the model, the parameter τ_dopa_ = 10 ms representing the time constant of the phasic effect of DA on corticostriatal learning (Equation 11) was artificially set to a very fast value to ensure that learning occurs at the very beginning of the burst. Slower values led to the situation where NAcc could only predict the occurrence of reward delivery at the end of the burst, what arrives too late to effectively cancel the burst. In the model of Chorley and Seth (2011), bursting behavior occurs in a time window of 50 ms, which, coupled to the precise timing properties of STDP when compared to Hebbian learning rules, allows a very sharp learning of the time elapsed since CS onset. How can very high oscillation frequencies (the original Striatal-Beat Frequency model uses oscillators in the delta range 8–13 Hz) accommodate with such large DA bursts is still an unresolved question.

In section 3.2, the earlier delivery of a reward lead to a VTA burst of the same amplitude as an expected reward, but not to a dip at the expected time, as observed experimentally (Hollerman and Schultz, 1998). This is only because the CS representation stops when the US disappears. If the CS were maintained for a longer duration, such a dip would in fact be observed as the oscillators in vmPFC would still signal the elapsed duration. There is a need for a reset mechanism stopping the oscillators at reward delivery. A possible pathway would involve a closed-loop between vmPFC and the ventral BG, with the inhibitory projection from VP on the mediodorsal nucleus of the thalamus (MD) being able to stop thalamo-cortical oscillations between MD and vmPFC at reward delivery. The problem of resetting temporal representations after reward delivery is common to many models (see Daw et al., 2006 for a review), at the notable exception of the model of Ludvig et al. (2008).

Although successfully reproducing the known effects of reward magnitude on DA activity, the proposed model does not investigate the case where less reward than expected, instead of no reward at all. Experimentally, VP gets activated by large rewards and inhibited by small ones (Tachibana and Hikosaka, 2012), while LHb shows the opposite pattern (Hikosaka et al., 2008). Based on the current model, we propose that the comparison between predicted and received reward may be computed in VP through the competition between inhibitory inputs from NAcc and excitatory inputs from PPTN and is further transmitted to VTA either directly or through disinhibition of LHb and RMTg. A further refinement of the model in these areas may also shed some light on the influence of aversive stimuli, which are able to activate the lateral habenula and produce DA dips (Matsumoto and Hikosaka, 2007) but also to generate bursts in some subpopulations of VTA (Brischoux et al., 2009; Lammel et al., 2012).

The subthalamic nucleus (STN) has been left out of the model, although it is part of the ventral BG. Like NAcc, its medial part receives cortical inputs from the medial prefontal cortex, but it projects excitatorily on the part of VP receiving connections from the core of NAcc. It has been shown to encode both reward magnitude, reward expectation and errors (Darbaky et al., 2005; Lardeux et al., 2009) and is important for Pavlovian-to-Instrumental transfer of learning (Winstanley et al., 2005). STN may signal the motivational value of stimuli to VP, complementing the information received from PPTN. Future extension of this model to instrumental learning will have to investigate the role of STN more deeply.

Similarly, the cerebellum is a very important player in aversive conditioning, as in the eyeblink conditioning paradigm (Christian and Thompson, 2003; Thompson and Steinmetz, 2009). It has been left out of the model as its involvement in appetitive conditioning is still unknown. However, it is now acknowledged that the cerebellum and the basal ganglia communicate more with each other than initially thought: in particular, the cerebellum projects on thalamic nuclei which directly contact the striatum, especially the D2-type neurons of the indirect pathway (Bostan and Strick, 2010). How the BG and the cerebellum cooperate during conditioning still has to be explored.

The role of the ventral striatum in timing processes is also subject to debate. Several studies have shown that NAcc plays no important role in the timing of instrumental responding (Meck, 2006; Galtress and Kirkpatrick, 2010), contrarily to the timing of Pavlovian responses (Singh et al., 2011). However, both processes are interrelated, as they both rely on dopaminergic activation, while NAcc is considered as a crucial site for Pavlovian-to-Instrumental transfer of learning (Corbit and Balleine, 2011). The Striatal-Beat Frequency model was initially proposed for the timing of instrumental responses, and identified the dorsal striatum as a potential substrate for the coincidence detection. Are two sites of temporal learning really needed for such interdependent processes? Kirkpatrick (2013) proposed a functional model of the interactions of timing and prediction error learning, where NAcc and BLA cooperate to compute the reward value, while the timing of the association itself is learned in the dorsal BG and transmitted to the DA system through its output GPi (internal segment of the globus pallidus). Indeed, the border regions of GPi, which is usually considered as composed of GABAergic neurons projecting to the thalamus, have been shown to send an excitatory projection on LHb, what can in turn produce DA dips (Hong and Hikosaka, 2008). These LHb-projecting neurons in GPi exhibit a negative reward-prediction error pattern, excited by reward omission and inhibited by large rewards, which is similar to the one in LHb but occurs slightly in advance. These border regions of GPi receive projections from both the dorsal and ventral striatum, so it is possible that both the dorsal and ventral parts of the BG cooperate to learn the temporal properties of both action-outcome and stimulus-reward associations.

The proposed model is also rather conservative regarding the role of the amygdala in timing: given that the amygdala is a key structure in acquiring, processing and storing Pavlovian associations and that timing is a fundamental component of conditioning, there should be some neural correlates of temporal processing in the amygdala. Several lines of evidence indeed suggests such an involvement, as reviewed in Díaz-Mataix et al. (2013). In particular, a subgroup of neurons in BLA exhibits a strong change in firing rate at the time when the US is expected but not delivered (Belova et al., 2007), while some others show anticipatory activity for the reward, proportional to the instantaneous reward delivery probability (Bermudez and Schultz, 2010). This phenomenon might be particularly relevant for extinction, where the prolonged absence of the US should decrease the conditioning strength associated to the CS (Tye et al., 2010). The question is now from where does this timing information come from. Is it only signaled by the dopaminergic projection from VTA to BLA, which is able to modulate both firing and learning in BLA, or do other structures such as the hippocampus or vmPFC play a role?

In our model, the CS-related bursts in VTA arise from the BLA → CE → PPTN pathway, both during and after learning. However, CE has been shown to be important for learning but not expressing approach to appetitive cues (McDannald et al., 2004; Groshek et al., 2005). One possibility is that associations learned in the amygdala are progressively transferred to the orbitofrontal or ventromedial prefrontal cortices, which are known to project excitatorily onto VTA (Geisler et al., 2007). It is indeed known that frontal-amygdalar interactions are necessary for the formation and use of expectancies of reinforcers in the guidance of goal-directed behavior (Holland and Gallagher, 2004). It is therefore possible that the value associated to a reward is first associated to the sensory features of the predicting CS in the amygdala (what can initially generate CS-related bursts) but that the prefrontal cortex progressively learns to compute the motivational value of the CS and activate the dopaminergic system with this information. The known inhibitory projection from the medial prefrontal cortex to BLA might provide a direct mechanism to implement this transfer of responsability (Carmichael and Price, 1995), while NAcc is at a central position to control their interplay (O'Donnell and Grace, 1995).

## 5. Conclusion

We have proposed a neuro-computational model linking reward processing to timing processes by focusing on the observed activity patterns of dopaminergic neurons during Pavlovian conditioning. We isolated a group of brain areas involved in the different aspects of appetitive conditioning and built a network using known anatomical connections. The resulting neural network model reproduces several experimental observations, while providing a robust mechanism for classical conditioning which can be implemented on a robotical platform. Its structure provides a first step toward building biologically realistic models of instrumental responding by understanding how the dopaminergic signal can be generated. Future extensions of this model, especially by focusing on the ventral BG and the crucial role of NAcc, will allow to learn the motivational value of different stimuli by transferring the value of an outcome to the action associated to the stimulus. They will ultimately allow to study the neural substrates of goal-directed behavior and their relationship with neuromodulators such as dopamine.

### Conflict of interest statement

The authors declare that the research was conducted in the absence of any commercial or financial relationships that could be construed as a potential conflict of interest.

## Footnotes

^1^http://www.tu-chemnitz.de/informatik/KI/projects/ANNarchy/index.php
